# Repeated Inoculation of Young Calves With Rumen Microbiota Does Not Significantly Modulate the Rumen Prokaryotic Microbiota Consistently but Decreases Diarrhea

**DOI:** 10.3389/fmicb.2020.01403

**Published:** 2020-06-24

**Authors:** Dengpan Bu, Xin Zhang, Lu Ma, Tansol Park, Lingling Wang, Mengzhi Wang, Jianchu Xu, Zhongtang Yu

**Affiliations:** ^1^State Key Laboratory of Animal Nutrition, Institute of Animal Science, Chinese Academy of Agricultural Sciences, Beijing, China; ^2^CAAS-ICRAF Joint Lab on Agroforestry and Sustainable Animal Husbandry, Beijing, China; ^3^Hunan Co-Innovation Center of Animal Production Safety, CICAPS, Changsha, China; ^4^College of Animal Science and Technology, Yangzhou University, Yangzhou, China; ^5^Department of Animal Sciences, The Ohio State University, Columbus, OH, United States; ^6^Kunming Institute of Botany, Chinese Academy of Sciences, Kunming, China

**Keywords:** calves, diarrhea, metataxonomics, oral inoculation, rumen microbiota

## Abstract

The complex rumen microbiota exhibits some degree of host specificity. The undeveloped simple rumen microbiota is hypothetically more amendable. The objective of this study was to investigate if the rumen prokaryotic microbial assemblage of young calves can be reprogrammed by oral inoculation with rumen microbiota of adult cows. Twenty newborn male calves were randomly assigned to four groups (*n* = 5 per group), with two groups being orally inoculated with rumen microbiota (fresh rumen fluid) collected from two lactating dairy cows, while the other two groups receiving autoclaved rumen fluid collected from another two donor cows. Each calf was orally drenched with 100, 200, 300, 400, and 500 mL of the rumen fluid at d3, d7, d21, d42, and d50, respectively, after birth. The inoculation with rumen microbiota did not affect (*P* > 0.05) feed intake, average daily gain (ADG), heart girth, or feed conversion ratio but significantly (*P* < 0.01) lowered instance of diarrhea. At the age of 77 days (27 days post-weaning), all the calves were slaughtered for the sampling of rumen content and determination of empty rumen weight. Rumen fermentation characteristics were not affected (*P* > 0.05) by the inoculation. Rumen prokaryotic microbiota analysis using metataxonomics (targeting the V4 region of the 16S rRNA genes) showed that the calf rumen prokaryotic microbiota differed from that of the donors. Two *Succinivibrionaceae* OTUs, two *Prevotella* OTUs, and one *Succiniclasticum* OTU were predominant (relative abundance > 2%) in the donors, but only one *Succinivibrionaceae* OTU was found in the calves. On the other hand, five other *Prevotella* OTUs were predominant (>3%) in the calves, but none of them was a major OTU in the donors. No correlation was observed in relative abundance of major OTUs or genera between the donor and the calves. Principal coordinates analysis (PCoA) based on weighted UniFrac distance showed no significant (*P* > 0.05) difference in the overall rumen prokaryotic microbiota profiles among the four calf groups, and principal component analysis (PCA) based on Bray-Curtis dissimilarity showed no significant (*P* > 0.05) difference in functional features predicted from the detected taxa. Nor the calf rumen microbiota showed any clustering with their donor’s. Repeated oral inoculation with rumen microbiota probably has a limited effect on the development of rumen microbiota, and the rumen microbiota seems to develop following a program determined by the host and other factors.

## Introduction

The species composition and structure and the associated functions of the rumen microbiota determine the most important production phenotypes of ruminants, including feed utilization efficiency, nitrogen efficiency, the output of wastes including nitrogen and methane, and host health (Kittelmann et al., 2014; Li and Guan, 2017; Sasson et al., 2017; Paz et al., 2018). It has been a long-standing pursuit to modulate the rumen microbiome to effectively improve animal production performance (Parker, 1990; Jouany, 1994; Patra and Saxena, 2009; Yanez-Ruiz et al., 2015; Clemmons et al., 2019). Most of the manipulation efforts were made using dietary interventions, and they were met with mixed success (Patra and Saxena, 2009; Clemmons et al., 2019). Additionally, because of the rumen microbiota is rather stable and resilient (Weimer, 2015), all dietary interventions need to be continuous, thus increasing feeding cost. Furthermore, adverse effects including lowered feed intake and digestion often result at effective doses (Patra and Yu, 2014). Although from a microbiological perspective, rumen microbes of desired features can be inoculated into a rumen to improve certain aspects of the rumen function, such as fiber digestion, inoculation of the rumen of adult ruminants with *Ruminococcus albus* and *R. flavefaciens* did not enhance fiber digestion (Krause et al., 2001), while *Megasphaera elsdenii* inoculation did not attenuate subacute ruminal acidosis (SARA) (Klieve et al., 2003; Zebeli et al., 2012; Arik et al., 2019). Inoculation of dairy cows that exhibited milk fat depression with whole rumen content from non-milk fat-depressed cows only slightly accelerated the recovery of *de novo* synthesis of fatty acids (FA) in the former (Rico et al., 2014). A recent study showed that replacing 70% of the rumen content of steers with that of basins altered the rumen microbiota of the recipients and rumen metabolism and improved nitrogen utilization, but the expected improvement in fiber digestion was never observed (Ribeiro et al., 2017). Recent studies also demonstrated that ruminants have considerable control over its rumen microbiota, and particularly the rumen microbiota of adult dairy cows is rather resilient (Weimer et al., 2010, 2017). Colonization resistance and resilience of the rumen microbiota in mature ruminant animals negate manipulation of their rumen microbiota. Therefore, the early life of young ruminants represents an opportunity to modulate the rumen microbiome to achieve potential lasting effects in adult ruminants (Yanez-Ruiz et al., 2015).

Newborn ruminant animals including calves are born with a sterile non-functional rumen (Soares et al., 1970; Alexander and Lysons, 1971; Baldwin et al., 2004), which is colonized gradually with a diverse assemblage of microbes primarily anaerobic bacteria after birth (Rey et al., 2014; Guzman et al., 2015; Dias et al., 2018). From a perspective of microbial ecology, the young rumen ecosystem is more amendable because its very simple microbiota has much less colonization resistance and resilience than that of adult animals. Indeed, early studies showed that inoculated individual bacteria could colonize and persist in the rumen of gnotobiotically-reared lambs (Fonty et al., 1997) and meroxenic lambs (Fonty et al., 1983), both of which had a very simple rumen microbiota. Early dietary interventions were also shown to be more effective in modulating the rumen microbiota development in dairy calves although the long-term impacts on milk production (Dill-McFarland et al., 2019) or the desired effects (CH_4_ abatement to be exact) did not last after the intervention ceased (Saro et al., 2018). Early studies in the mid-1900s used either ruminal fluid or content from adults to inoculate calves to better understand early rumen microbial establishment, and they showed that the inoculation accelerated the establishment of rumen protozoa but had no effect on feed intake, weight gain, rumen pH, or predominant culturable bacteria typical of mature cattle (Pounden and Hibbs, 1950; Bryant and Small, 1960). One study also showed that faunation via oral gavage of protozoa-enriched rumen fluid from a bull at 5 and 6 weeks of age faunated the rumen of pre-weaned Holstein calves and increased methane production, rumen empty weight, rumen pool size of nitrogen, and crude fat with no effect on intake of ME per kg dry matter (DM) or animal growth (Schonhusen et al., 2003). A very recent study found no effects of oral inoculation of pre-weaned calves with a bacteria-enriched or a protozoa-enriched inoculum on animal growth or rumen bacterial microbiota (Cersosimo et al., 2019). The objective of the present study was to examine if repeated (five times), incremental (100 to 500 ml each time), and early (at d 3 of age) oral inoculation of pre-weaned calves with adult rumen microbiota on animals (feed intake, growth, intestinal development, and diarrhea) and their rumen microbiota post-weaning (27 days post-weaning). The new information on rumen microbiota can help understand the lack of desirable effect on the growth performance of pre-weaned calves when they were inoculated with rumen microbiota from adult cattle.

## Materials and Methods

### Animal Experiment and Rumen Microbiota Inoculation

The handling and care of the animals followed a protocol (protocol number: IAS2018115) approved by the Institutional Animal Care and Use Committee (IACUC) of the Institute of Animal Science, Chinese Academy of Agricultural Sciences.

Twenty newborn male Holstein calves with initial body weight (BW) between 38 and 50 kg were procured from a local dairy breeding farm. They were randomly assigned to four groups (*n* = 5 per group) in two treatments, with one treatment (Inoc) being oral inoculation with rumen microbiota (fresh rumen fluid) collected from two donor cows, while the other treatment (Ctrl) being oral inoculation with autoclaved rumen fluid collected from another two donor cows. The two treatments were balanced with initial BW. Right after birth, all the calves were each fed 4 L of colostrum (kept at 48°C, Brix percentage ≥ 20) within the first hour after birth. Colostrum IgG was tested using an optical refractometer (HT113ATC; Httechltd Tianyuan Optical Instruments Co., Ltd., China). Only colostrum with an IgG level ≥ 50 mg/mL was fed to the calves. The calves were then transferred to individually fenced calf hutches (1.5 × 2.0 m) between 12 and 18 h after birth. The hutches had sand bedding and were on the breeding farm. The hutches were disinfected before the arrival of the calves.

The calves were fed pasteurized whole milk twice daily at 0800 and 1500, from bottles from d 1 to 3 and then from individual buckets. On average, the milk contained 12.3% DM, 3.6% crude protein (CP), and 4.1% ether extract. Bottles or buckets were cleaned immediately after each use. To meet the increasing nutritional requirement as the calves grew, the calves were fed increasing amounts of milk daily: 2 L from d 1 to 7 and 3.5 L from d 8 to 42. From d 43 to 49, the daily milk allowance gradually decreased to 2 L with equal daily decrement, and all the calves were completely weaned off milk at d 50. All the calves had *ad libitum* access to fresh drinking water and a pelleted starter feed (offered twice daily at 0900 and 1800) from d 3 to 77. The starter feed contained 89.5% DM, which had 20% CP, 2.8% ether extract, 6.7% ash, 1.0% calcium, 0.5% phosphorus, 10.0% acid detergent fiber (ADF), and 18% neutral detergent fiber (NDF). No forage was offered to the calves because feeding hay intake can decrease solid feed intake and calf growth (Hill et al., 2019; Engelking et al., 2020). Daily starter feed intake was recorded. All calves were slaughtered at d 77 for the sampling of rumen content.

Four lactating Holstein cows fitted with permanent rumen cannula were used as the donors of rumen fluid, with each donor providing rumen fluid for one of the four groups (*n* = 5) of calves throughout the entire experiment. Two donors (referred to as Inoc donors) provided fresh rumen fluid for the two Inoc groups of calves (donors A and B for Inco-A and Inoc-B, respectively), while the other two donors provided rumen fluid for the two Ctrl groups of calves (donors C and D for Ctrl-A and Ctrl B, respectively). The donor cows had similar parity and days in milk (DIM) and were fed the same diet and housed in the same barn. The rumen fluid from the two Inoc donors was collected before the morning feeding on each inoculation day, strained through two layers of sterile cheesecloth, and kept at 39°C in a sealed thermos until inoculation at morning feeding (within 1 h). The rumen fluid from the two Ctrl donors was also collected before the morning feeding but one day before each inoculation day, strained through two layers of sterile cheesecloth, autoclaved at 121°C for 30 min, and kept at 39°C in a sealed thermos until inoculation at morning feeding the next day (this allowed both the Inoc and the Ctrl calves to be inoculated on the same day). Each calf was orally drenched with 100, 200, 300, 400, and 500 ml of respective rumen fluid at 3, 7, 21, 42, and 50 days of age, respectively. One aliquot of each rumen fluid sample collected from the two Inoc donors was preserved at −80°C until DNA extraction.

### Animal Growth Measurement, Sampling, and Analysis

Starter offered and refused amounts were recorded daily for each calf. Starter feed samples and refusals were collected daily and individually mixed thoroughly after drying and ground to pass through a 1-mm screen using a Wiley mill (Arthur H. Thomas Co) before the samples were analyzed for content of NDF and ADF (Van Soest et al., 1991), CP (method 984.13; AOAC, 1990) and ether extract (method 920.39; AOAC, 1990). Ash content was determined by incineration in a muffle furnace at 550°C. Milk samples were analyzed for total solids using an infrared analyzer MilkoScan^TM^ FT 120 (FOSS Analytical A/S, Hillerød, Denmark). Bodyweight, shoulder height, body length, heart girth, and cannon bone circumference were measured immediately after birth and thereafter weekly until the end of the experiment. Fecal consistency was scored daily on a scale from 0 to 3, with 0 = normal consistency, 1 = semi-formed or pasty, 2 = loose feces, and 3 = watery feces. Calves with a fecal score ≥ 2 were considered positive for diarrhea (McGuirk, 2008).

All the calves were slaughtered at day 77. The rumen content was emptied into individual foil trays and mixed, and the pH was immediately measured using a portable pH meter. Rumen digesta samples were collected and aliquots were stored at −20°C until further analysis. The VFA profiles of the rumen samples were analyzed using gas chromatography (Zhou et al., 2011). Ammonia nitrogen concentration was determined colorimetrically using Berthelot’s reagent (Chaney and Marbach, 1962; Rhine et al., 1998).

### Rumen Prokaryotic Microbiota Analysis

Genomic DNA was extracted from the rumen samples of the calves and the donors using the RBB + C method (Yu and Morrison, 2004). The quality of the DNA samples was evaluated using agarose (0.8%) gel electrophoresis, while the concentrations were quantified using a Quant-iT^TM^ dsDNA Assay Kit (Invitrogen Corporation, Carlsbad, CA, United States). The rumen prokaryotic microbiota represented by the rumen DNA samples was analyzed for diversity and composition as described previously using metataxonomics (Park and Yu, 2018). Briefly, dual indexed 16S rRNA gene amplicon libraries for both bacteria and archaea were prepared using primers 515F (5′-GTGCCAGCMGCCGCGGTAA-3′) and 806R (5′-GGACTACHVGGGTWTCTAAT-3′) (Caporaso et al., 2011) with a unique barcode for each DNA sample. The amplicon libraries were sequenced using the 2 × 300 paired-end protocol on the Illumina MiSeq platform. After demultiplexing of the amplicon sequences, the barcode and primer sequences are trimmed off using Cutadapt (Martin, 2011). The built-in plugins within QIIME2 (version 2019.4) (Bolyen et al., 2019) were used to analyze the amplicon sequences. First, the DADA2 plugin was used to denoise the forward and reverse reads by filtering out low-quality (*Q* < 25) reads and to merge them followed by chimera removal (Callahan et al., 2016). The raw amplicon sequence data are available in the NCBI sequence read archive (SRA) under the accession number PRJNA562303.

Amplicon sequencing variants (ASVs) were assigned to taxa using the naïve Bayesian taxonomic classifier (Wang et al., 2007) and a pre-trained Greengenes (13_8 version) reference database that was trimmed to retain only the V4 region. The ASVs that matched to mitochondria or chloroplast (seven ASVs that accounted for 0.072% of the total sequences) were filtered out. Major classified phyla, families, and genera, each of which was detected in at least 50% of the rumen samples were discussed in this study. Alpha diversity of each sample was examined with respect to species richness, evenness, Faith’s phylogenetic diversity, Shannon diversity index, and Simpson index based on the rarefied ASV tables using 8,170 ASVs per sample. Principal coordinates analysis (PCoA) was computed based on weighted UniFrac distance matrices to compare the overall dissimilarity of prokaryotic microbiota among different groups of rumen samples. Differentially abundant taxa between the Ctrl and the Inoc calves were identified using linear discriminant analysis (LDA) effect size (LEfSe) implemented in the Galaxy online tool^1^ with an LDA score of two as cutoff (Segata et al., 2011). The number of taxa shared by and exclusively found in individual groups of rumen samples was visualized using the R package Venn Diagram (Chen and Boutros, 2011). Relative abundances of major taxa at phylum, family, and genus level in each group of rumen samples were visualized using the QIIME2 feature-table heatmap plugin.

Amplicon sequencing variants and the corresponding BIOM table were used to predict functional features from the 16S rRNA gene sequence data using PICRUSt2 (Douglas et al., 2020) with the default options. Briefly, the input ASVs were aligned with the reference sequence alignment followed by placing these sequences into reference phylogeny to build a tree file. Then, gene family abundances were predicted using the tree file as input with pre-calculated gene- or function-count tables as the reference. The abundance of individual gene families was normalized with the 16S copy number of each ASV, and the normalized abundance was used to determine the predicted functional profiles per sample using ASVs’ abundance BIOM table. Principal component analysis (PCA) was computed based on Bray-Curtis dissimilarity to compare the overall functional profiles based on the predicted KEGG orthologs among the different groups of rumen samples. The PCA plots were visualized using the R package ggfortify (Tang et al., 2016). LEfSe was used to identify differentially abundant KEGG pathways and modules with an LDA score of two as the cutoff.

### Statistical Analysis

Except for feed conversion ratio, data on starter intake, and growth measurements were analyzed using a repeated-measures mixed model (PROC MIXED) of SAS 9.4 (SAS Institute Inc., Cary, NC, United States), with calf as the random effect and treatment, age (wk), their interaction as fixed effects, and autoregressive (order 1) being assumed for the covariance structure. The initial calf BW was included in the model as a covariate for the statistical analysis of final body weight. The data were presented as least squares means, and the difference between the two treatments was tested using *F*-test. The diarrhea data were analyzed using the Chi-square test (Proc Freq) of SAS 9.4. Significance was declared at *P* < 0.05.

Alpha diversity data of the rumen microbiota and the predicted gene counts were analyzed using the GLIMMIX procedure of SAS 9.4 (SAS Institute Inc., Cary, NC, United States). Permutational multivariate analysis of variance (PERMANOVA) was used to evaluate the PCoA clustering of the sample groups using the QIIME2 plugin. For PCA plots with predicted KEGG-orthologs, PERMANOVA was used to evaluate the overall dissimilarity of functions between the Ctrl and the Inoc calves with 9,999 permutations using PAST3 software (Hammer et al., 2001).

## Results

### Repeated Inoculation With Rumen Microbiota Had No Effect on Animal Growth Performance

At the end of the experiment, no significant difference was noted in any of the measurements of feed intake, feed conversion ratio, or animal growth including BW, ADG, body height, body length, heart girth, or cannon bone circumference between the Inoc and the Ctrl calves (Table 1). The weekly average of the above measurements was also similar between the Inoc and the Ctrl calves and between the two groups of each treatment (data not shown). The above measurements increased as the calves grew, but no significant treatment by week interactions was noted. The inoculation did not affect the pH and concentrations of total VFA or individual VFA, or the ammonia-N concentration in the rumen (data not shown).

**TABLE 1:** Summary of calf growth measurements of the inoculated calves (Inoc) and control calves (Ctrl).

	Treatment		SEM^ 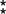 ^	*P*-value^ 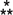 ^		
	Ctrl*	Inoc*		Trt^♣^	Week	Trt × Week
Starter DMI of the last week (kg/d)	2.07	2.16	0.17	0.71	<0.01	0.24
DMI (kg/d) over the experiment (kg/d)^#^	1.09	1.11	0.06	0.68	<0.01	0.51
Starter DMI (kg/d) over the experiment	0.62	0.65	0.06	0.68	<0.01	0.51
Body weight (kg)	97.4	95.7	3.33	0.73	<0.01	0.52
Average daily gain (kg/d)	1.01	0.97	0.17	0.47	<0.01	0.60
Body height (cm)	93.5	93.2	0.57	0.71	<0.01	0.08
Body length (cm)	93.6	95.0	0.84	0.23	<0.01	0.93
Heart girth (cm)	97.1	96.7	1.68	0.93	<0.01	0.83
Cannon bone circumference (cm)	13.0	13.1	0.19	0.66	<0.01	0.18
Feed conversion ratio (d 0 – d 77)	1.51	1.54	0.06	0.76	–^ 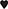 ^	–^ 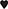 ^

** Ctrl, control calves receiving autoclaved rumen fluid; Inoc, calves receiving fresh rumen fluid. ^
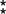
^ standard error of mean. ^
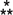
^ calculated from *F*-test. ^#^ of both milk and starter intake over the experiment. ^♣^ treatment effect: inoculated with autoclaved rumen fluid (Ctrl) or fresh rumen fluid (Inoc). ^
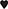
^ calculated over the course of the experiment, and thus no temporal response or treatment × week interaction.*

### Repeated Inoculation With Rumen Microbiota Reduced Diarrhea

Compared to the Ctrl calves, the Inoc calves had significant (*P* < 0.01) less diarrhea, in terms of overall incidences and frequency, and incidences and frequency both before and after weaning (Table 2). Larger decreases in diarrhea incidences and frequency were also observed before weaning (decreased by 45.5 and 50.9%, respectively) than after weaning (decreased by 33.3 and 11.0%, respectively).

**TABLE 2:** Effects of inoculation with fresh rumen fluid on incidence of diarrhea in calves.

	Treatment		*P*-value^ 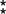 ^
	Ctrl*	Inoc*	
Overall incidence of diarrhea^†^	91	69	<0.01
Overall diarrhea frequency^#^	13.13	8.96	<0.01
Incidence of diarrhea before weaning	66	36	<0.01
Diarrhea frequency before weaning	14.97	7.35	<0.01
Incidence of diarrhea after weaning	33	25	>0.05
Diarrhea frequency after weaning	11.79	9.92	>0.05

*^∗^Ctrl, control calves receiving autoclaved rumen fluid; Inoc, calves receiving fresh rumen fluid. ^
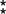
^ calculated from Chi-square test. ^†^ number of days × number of calves with a fecal score ≥ 2. ^#^ incidence of diarrhea/(total number of calves × duration (days) of study duration).*

### Repeated Inoculation With Rumen Microbiota Had Limited Effect on the Rumen Prokaryotic Microbiota and Its Functions in Calves

**FIGURE 1:**
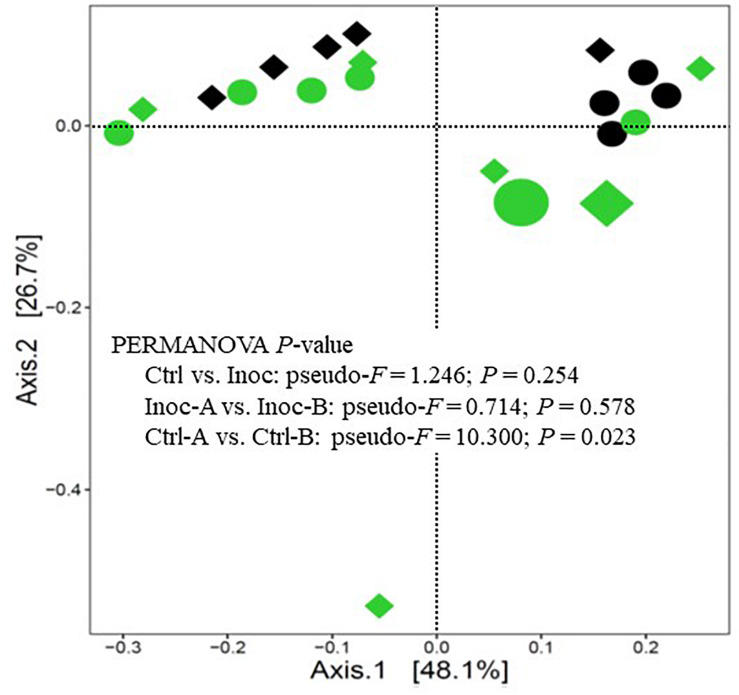
PCoA plot showing the overall comparison of the rumen prokaryotic microbiota. Large green circle, Inoc-A donor; small green circles, Inoc-A calves; large diamond, Inoc-B donor; small green diamonds, Inoc-B calves; small black circles, Ctrl-A calves; small black diamonds, Ctrl-B calves. Data of the donors for the control calves were not shown.

On average, 100,183 ± 54,887 prokaryotic sequences were obtained per sample though fewer sequences were generated from the donor samples (Supplementary Table S1). The calculated Good’s coverage exceeded 99% for all the samples. As expected, the donor prokaryotic microbiota was more diverse and had a higher richness than that of the calves (Table 3). As shown on the PCoA scatter plot (Figure 1), the two donors of the two groups of Inoc calves had numerically different (statistical analysis could not be done because only one donor was used for each inoculation group) prokaryotic microbiota. The differences were also reflected in the relative abundance of the major phyla, families, and genera (Supplementary Figure S1). At the genus level, the Inoc-B donor appeared to have less *Shuttleworthia* and *Ruminococcus*, but more *Sharpea*, *Methanobrevibacter*, *Desulfovibrio*, and *Pyramidobacter* than the Inoc-A donor (Supplementary Figure S1C).

**TABLE 3:** Summary of alpha-diversity measurements of the rumen microbiota.

Diversity measurements	Donor^†^	Treatment				SEM^ 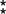 ^	*P*-values^ 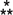 ^	
		Ctrl*		Inoc*				
		Ctrl-A	Ctrl-B	Inoc-A	Inoc-B		Trt^♣^	Donor^†^
Observed ASVs	370	106	77	104	117	9.43	0.283	0.690
Chao1	377	125	92	122	140	12.63	0.353	0.652
Evenness	0.86	0.58	0.51	0.48	0.54	0.02	0.446	0.367
Faith’s phylogenetic diversity	23.23	9.10	5.79	7.92	9.39	0.64	0.288	0.429
Shannon index	7.30	3.84	3.18	3.24	3.62	0.14	0.884	0.375
Simpson index	0.98	0.81	0.75	0.74	0.79	0.02	0.869	0.474

**Ctrl, control calves receiving autoclaved rumen fluid; Inoc, calves receiving fresh rumen fluid. ^
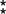
^ standard error of mean. ^
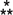
^ calculated using *F*-test. ^♣^ treatment effect: inoculated with autoclaved rumen fluid (Ctrl) or fresh rumen fluid (Inoc). ^
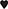
^ calculated based on the two groups of Inoc calves (Inoc-A and Inoc-B). ^†^ Each group of calves received rumen fluid from only one of four donor cows throughout the experiment. Only the sequence data of the donors for the included calves were presented. Good’s coverage exceeded 99% for all the samples (data not shown).*

Overall, the donors had numerically more (statistical analysis could not be done because only one donor was used for each inoculation group) phyla, families, and genera than both the Ctrl calves and the Inoc calves (Supplementary Figure S2). No significant difference (*P* > 0.05) in the common alpha diversity measurements was evident between the Inoc and the Ctrl calves or between the two groups of Inoc calves (Inoc-A vs. Inoc-B) (Table 3). The PCoA showed no significant difference (*P* > 0.05) in the overall rumen prokaryotic microbiota between the Inoc and the Ctrl calves. No significant difference was observed either between the two groups of Inoc calves, but the two Ctrl groups had different overall prokaryotic microbiota (*P* < 0.05). The donors and their respective Inoc calves did not have more prokaryotic phyla, families, or genera shared than the non-corresponding pairs (Inoc-A donor vs. Inoc-B calves, or Inoc-B donor vs. Inoc-A calves) (Supplementary Figure S3). The two groups of Inoc calves did not share more taxa compared to the two Ctrl groups (Supplementary Figure S4).

**FIGURE 2:**
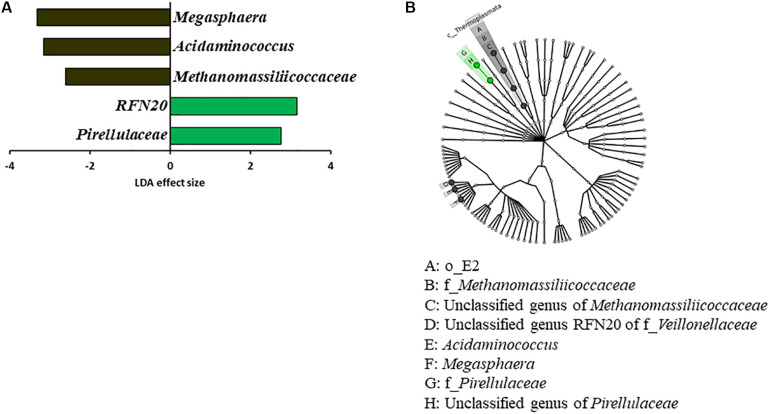
LEfSe plots showing **(A)** the families and genera of bacteria and archaea enriched among the Ctrl calves (black) vs. among and the Inoc calves (green) and **(B)** the corresponding taxonomic cladogram of the enriched taxa.

Some OTUs were found in both the two donors and the two groups of inoculated calves, which were not found in the two control calf groups (Supplementary Table S2). These include two OTUs (ID: 17 and 25) only assignable to the order *Bacteroidales*, one *Clostridiales* OTU (ID: 44), three *Ruminococcaceae* OTUs (ID: 117, 121, 124), three OTUs assigned the candidate family S24-7 (ID: 128, 139, 148), six *Prevotella* OTUs (ID: 237, 408, 417, 457, 461, 467), one *Ruminococcus* OTU (ID: 488), and one *Succinivibrio* OTU (ID: 520). Eighteen OTUs were found across all the donors and the calves irrespective of the inoculation. Comparison between the Inoc and the Ctrl calves using LEfSe identified significant enrichment of *Megasphaera*, *Acidaminococcus*, and *Methanomassiliicoccaceae* among the Ctrl calves, while RFN20 (an uncultured genus within the family *Veillonellaceae*) and *Pirellulaceae* were significantly enriched in the Inoc calves (Figure 2). LEfSe analysis also showed that *Oscillospira* was enriched in the Inoc-A donor, whereas *Fibrobacter* was enriched in the Inoc-B donor. Compared to Inoc-A, Inoc-B had enriched *Bulleidia*.

**FIGURE 3:**
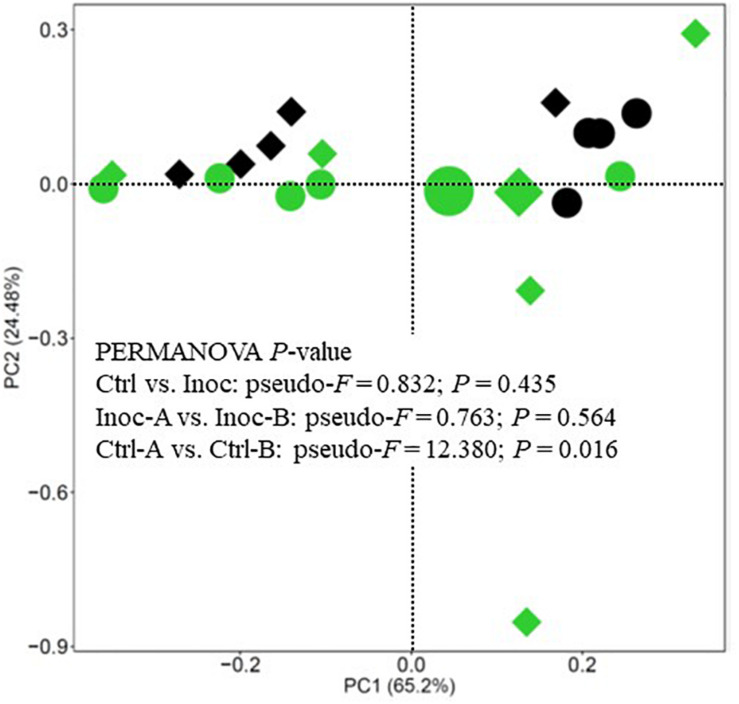
PCA plot showing the overall comparison of predicted functional features of the rumen microbiota (based on KEGG Orthology database). Large green circle, Inoc-A donor; small green circles, Inoc-A calves; large diamond, Inoc-B donor; small green diamonds, Inoc-B calves; small black circles, Ctrl-A calves; small black diamonds, Ctrl-B calves. Data of the donors for the control calves were not shown.

**FIGURE 4:**
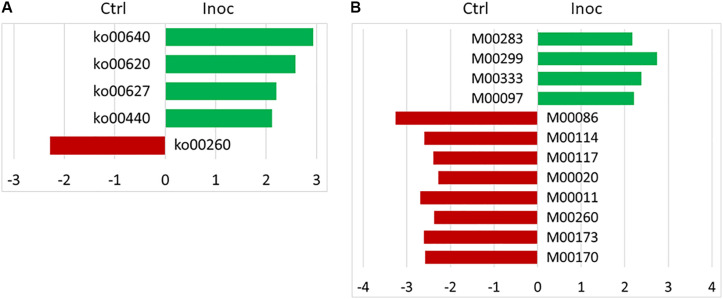
LEfSe plots showing the KEGG pathways **(A)** and KEGG pathway modules **(B)** enriched among the control (red) and the inoculated (green) calves. KEGG pathways: ko00640, propanoate metabolism; ko00620, pyruvate metabolism; ko00627, aminobenzoate degradation; ko00440, phosphonate and phosphinate metabolism; and ko00260, glycine, serine and threonine metabolism. KEGG pathway modules: M00283, PTS system, ascorbate-specific II component; M00299, spermidine/putrescine transport system; M00333, type IV secretion system; M00097, beta-carotene biosynthesis, GGAP ≥ beta-carotene; M00086, beta-oxidation, Acyl-CoA synthesis; M00114, ascorbate biosynthesis, plants, glucose-6P ≥ ascorbate; M00117, ubiquinone biosynthesis, prokaryotes, chorismate ≥ ubiquinone; M00020, Serine biosynthesis, glycerate-3P ≥ serine; M00011, citrate cycle, second carbon oxidation, 2-oxoglutarate ≥ oxaloacetate; M00260, DNA polymerase III complex, bacteria; M00173, reductive citrate cycle (Arnon-Buchanan cycle); and M00170, C4-dicarboxylic acid cycle, phosphoenolpyruvate carboxykinase type.

No significant difference in the number of functional features predicted against seven different databases (i.e., KEGG Orthology, COG, EC, PFAM, KEGG Module, KEGG pathway, and MetaCyc Pathways) was detected between the Ctrl calves and the Inoc calves or between the two groups of Inoc calves. The Inoc calves tended to have more functional features than the Ctrl calves when the KEGG pathway database was used in the functional prediction (Table 4). The overall predicted functional features were similar between the Ctrl calves and the Inoc calves (Figure 3). The two groups of Inoc calves did not differ in the overall predicted functional features, but the two Ctrl groups of calves differed. Analysis of the predicted functional features using LEfSe identified the KEEG pathways and pathway modules that were enriched in each treatment group of calves (Figure 4), with four pathways (propanoate metabolism, pyruvate metabolism, aminobenzoate degradation, and phosphonate and phosphinate metabolism) enriched among the inoculated calves, while one pathway (glycine, serine, and threonine metabolism) enriched among the control calves (Figure 4A). Some metabolic pathway modules were also found to be enriched differently between the two treatment groups (Figure 4B). However, the two groups of each treatment also exhibited different enrichment of both KEGG pathways and pathway modules (Supplementary Figure S5).

**TABLE 4:** Number of detected functional features of the rumen microbiome using by PICRUSt2.

Functional databases	Donor^†^	Treatment				SEM^ 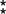 ^	*P*-values^ 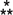 ^	
		Ctrl*		Inoc*				
		Ctrl-A	Ctrl-B	Inoc-A	Inoc-B		Trt^♣^	Donor^ 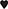 ^
KO	3,450	3,454	3,133	3,699	3,527	116.04	0.152	0.655
COG	3,116	3,080	2,801	3,217	3,077	74.07	0.138	0.528
EC	1,248	1,224	1,126	1,281	1,248	29.89	0.113	0.725
PFAM	4,514	4,322	3,944	4,528	4,398	111.89	0.120	0.707
KEGG-modules	229	223	208	227	223	3.66	0.155	0.760
KEGG-pathways	125	117	111	121	119	1.70	0.066	0.689
MetaCyc-pathways	260	260	238	268	261	6.04	0.177	0.707

** Ctrl, control calves receiving autoclaved rumen fluid; Inoc, calves receiving fresh rumen fluid. ^
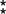
^ standard error of mean. ^
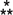
^ calculated using *F*-test. ^♣^ Treatment effect: inoculated with autoclaved rumen fluid (Ctrl) or fresh rumen fluid (Inoc). ^
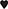
^ calculated based on the two groups of Inoc calves (Inoc-A and Inoc-B). ^†^ Each group of calves received rumen fluid from only one of four donor cows throughout the experiment. Only the sequence data of the donors for the included calves were presented.*

## Discussion

Numerous research efforts have been made to manipulate the rumen microbiome to increase feed utilization efficiency (Matthews et al., 2019); decrease the risk of rumen acidosis (Humer et al., 2018), methane emission (Haque, 2018), and nitrogen excretion in ruminants (Hartinger et al., 2018); and improve the nutrient composition of meat and milk (Toral et al., 2018). Mixed results from different studies and inconsistent responses from different animals within the same treatment groups challenge the practical application of the intervention strategies (Patra and Saxena, 2009; Clemmons et al., 2019). More promising results were obtained through dietary manipulation of the rumen microbiome of young animals (Saro et al., 2018; Dill-McFarland et al., 2019) than what can be achieved in adults. Early efforts to improve animal growth performance in pre-weaned calves did not result in appreciable benefits when they were inoculated with rumen microbiota from adult cattle (Conrad and Hibbs, 1953; Hardison et al., 1957; Hibbs and Conrad, 1958; Bryant and Small, 1960). Two recent studies also showed no significant improvement in animal growth performance or rumen fermentation characteristics (Schonhusen et al., 2003; Cersosimo et al., 2019). In the present study, we increased the inoculation frequency and inoculum volume. To determine the effect of the rumen microbes only, we also used autoclaved rumen fluid, rather than saline as the control. The results corroborate the general lack of desirable improvement in animal growth post-weaning, but the inoculation brought about unexpected benefits to gut health.

### Repeated Inoculation With Rumen Microbiota Had No Effect on Animal Growth Performance

The repeated inoculation did not significantly increase feed intake, feed conversion ratio, or any of the measurements of animal growth, either weekly or at the end of the experiment, which was 27 days post-weaning. These results are in general agreement with studies conducted decades ago (Hardison et al., 1957; Hibbs and Conrad, 1958; Bryant and Small, 1960; Schonhusen et al., 2003) and recent studies (Cersosimo et al., 2019; Yu et al., 2020b). We also found no effect of the inoculation on the fermentation characteristics (total VFA and individual VFA concentrations) in the rumen. The lack of stimulation to feed intake or rumen fermentation characteristics can better explain the null results observed in the inoculated calves. The microbes-free rumen fluid might also positively affect the animals. These results suggest that under typical farm conditions, inoculation of calves with rumen microbiota from adult cattle during the pre-weaning period, even starting at a very early age and repeatedly, probably yield limited benefits to their growth. However, future studies should also include control that is mock inoculated with saline.

### Repeated Inoculation With Rumen Microbiota Had Limited Effect on the Rumen Prokaryotic Microbiota and Its Predicted Functions in Calves

The rumen of newborn calves is believed to be sterile (Baldwin et al., 2004) and was hypothesized to be more amenable to colonization by externally introduced rumen microbes. However, the repeated inoculation only had limited effect on the overall diversity and composition of the rumen microbiota even though a few genera of bacteria were affected, which included significant increase in RFN20 (an uncultured genus within the family *Veillonellaceae*) and *Pirellulaceae* and decrease in *Megasphaera*, *Acidaminococcus*, and *Methanomassiliicoccaceae*. Some OTUs were also only found in the inoculated calves and the donor cows. The impact of the early inoculation on the predicted functions was also limited. Although the inoculated calves had the metabolic pathways of pyruvate and propionate enriched, no difference in total VFA or individual VFA concentrations were noted. Early studies in the 1900s found mixed results with respect to the colonization of culturable rumen bacteria even though they consistently found expedited establishment of rumen protozoa (Conrad et al., 1958; Bryant and Small, 1960). Bryant and Small (1960) reported little effect of inoculation with adult rumen microbiota on the time of the establishment of predominant culturable bacteria typical of mature cattle or on the total count of anaerobic bacteria. However, cud inoculation of pre-weaned calves coupled with feeding a pelleted high-roughage diet (hay to grain ratio, 3:1) increased the average rating (presence) of “Hay I indicator microorganisms (large G^+^ coccoids in closely-knit pairs),” but “Hay II microorganisms (three bacteria: large G^+^ square-ended rods, very large G^–^ cigar-shaped rods, and G^–^ short rods in fours and multiples of four)” were not detected (Conrad et al., 1958). Interestingly, the Hay II microorganisms were also found to be absent from the rumens of calves fed high grain rations (Pounden and Hibbs, 1948a; Hibbs et al., 1956) and in uninoculated calves kept away from older cattle (Pounden and Hibbs, 1948b). Unfortunately, the taxonomy of these “Hay microorganisms” was not determined.

Cersosimo et al. (2019) reported the first study to use metataxonomics in assessing the impact of inoculation (thrice, weekly at weeks 3 to 6) of pre-weaned calves with rumen bacteria (free of protozoa) or protozoa enrichment (still containing other rumen microbes). In the present study, we used the whole rumen microbiota from adult cows in inoculating the rumen of newborn calves starting at 3 days of age. We also increased the frequency of inoculation (five times, at d 3, d 7, d 21, d 42, and d 50) and the amounts of inoculum (100, 200, 300, 400, and 500 ml). Apparently, our inoculation approach did not significantly modulate the development of the rumen microbiota either under typical farm conditions, under which natural inoculation might have occurred. The lack of significant effect on rumen microbiota development mirrors the similar rumen fermentation characteristics (i.e., total VFA concentration, the molar proportion of individual VFAs, and ammonia concentration) and animal growth performance observed among the calves. It should be noted that we did not analyze the rumen microbiota at the end of weaning (d 50) or earlier, and thus it cannot be ruled out that the inoculation had impacted the rumen microbiota pre-weaning, but the changes diminished in the three weeks post-weaning. It is worth noting that we used autoclaved rumen fluid, which contained all the nutrients and fermentation products though some losses were expected, as the control. Future studies should also include a saline control to help determine if autoclaved rumen fluid can also influence rumen microbiota development.

The limited impact of inoculation of pre-weaned calves with adult rumen microbiota can be due to several reasons. First, pre-weaned calves are fed a very different diet than adult cattle, and thus the inoculated rumen microbes do not have the competitive advantage to colonize the young rumen. This explanation is consistent with the establishment of some of the bacteria characteristic of the rumen microbiota associated with hay ingestion in the inoculated calves fed alfalfa hay alone (Pounden and Hibbs, 1948b) and the increase of Hay I microorganisms in the inoculated calves fed a pelleted high roughage diet, while the Hay II microorganisms that were found in the rumen of calves fed high grain rations (Pounden and Hibbs, 1948a; Hibbs et al., 1956) and in uninoculated calves isolated from older cattle were missing (Pounden and Hibbs, 1948b). Following this line of reckoning, inoculation might be more effective if inoculation is coupled with a high-NDF starter or forage feeding pre-weaning and continues post-weaning when calves start to consume the diets fed to adult cattle. Second, individuality in gut microbiota composition is common as a result of multiple environmental and host genetic factors (Benson et al., 2010). The host does have substantial control over its rumen microbiota (Weimer et al., 2010, 2017), and such host effect, along with and stochastic colonization (Furman et al., 2020), can cause variation in the establishment of the rumen microbiota and thus diminishing the “inoculation effect.” Third, either the cultivation-based analysis in the early studies or the metataxonomic analysis using in recent studies and the present study could not identify the microbes to species or quantitatively assess the dynamics of microbial colonization. These analytical limitations can be addressed in future research to use whole metagenome shotgun (WMS) sequencing, which can help identify bacteria to species- even strain-level (Hillmann et al., 2018), or new improved pipeline that can help achieve species-level identification (Segota and Long, 2019), and synthetic spike-in standards, which can help quantify the abundance of individual taxa (Tourlousse et al., 2017). Nonetheless, the colonization process and the eventual diversity and composition of the rumen microbiota are determined by both deterministic and stochastic factors, but the relative importance of stochasticity versus determinism is unclear. The observed significant differences in the rumen microbiota and the predicted functions between the Ctrl groups, but not between the two Inoc groups, may suggest that the inoculation might have decreased the stochasticity of the rumen microbiota development. Integrated research combining multiple approaches, such as isolation of young calves from adult cattle, inoculation with defined microbiota, dietary treatments, and host genotyping, may help better define and understand the processes of the rumen microbiota development and the driving factors.

### Repeated Inoculation With Rumen Microbiota Reduces Diarrhea

Calf diarrhea is one of the most common and costly diseases in dairy herds. Based on a 2014 survey by the National Animal Health Monitoring System (NAHMS), 56.4% of calf mortality in the United States calves was attributed to diarrhea (USDA, 2014). Neonatal diarrhea also slows down animal growth, decreases reproductive performance and milk production later in lactations (Aghakeshmiri et al., 2017). Calf diarrhea is caused primarily by enteric bacteria or viruses (Foster and Smith, 2009). Weaning management (Richeson et al., 2012) and treatments using antimicrobials and non-antimicrobial alternatives (Lombard et al., 2015; Habing et al., 2016) are used to prevent and treat calf diarrhea. Although antimicrobials increase antimicrobial resistance among gut microbes, non-antimicrobials are not as efficacious as antimicrobials in the prevention and treatment of calf diarrhea. Unexpectedly, we found that the inoculation with adult rumen microbiota significantly decreased the frequency and duration of diarrhea among the inoculated calves. To the best of our knowledge, this is the first study to show that oral inoculation of pre-weaned calves with adult rumen microbiota can decrease calf diarrhea. The microbiological underpinnings of this unexpected finding, probably multifaceted, remains to be investigated in future studies, but the inoculation might have enhanced the development of the intestinal tract, microbiota, and gut immune and barrier functions as demonstrated in other studies (Bauer et al., 2006; Dhakal et al., 2019; Yu et al., 2020a). Indeed, two recent studies showed that inoculation of young lambs with rumen fluid from adult sheep increased the titers of IgA, SIgA, and IgG (Wu et al., 2016b) and intestinal mucosal immune system development (Wu et al., 2016a). Unfortunately, these authors did not report data on diarrhea or fecal microbiota.

Organic livestock producers require non-antimicrobial alternatives to meet the stringent USDA requirements that prohibit antimicrobial use on organically certified animals. Diarrhea is also very common among piglets (Song et al., 2015). Fecal microbiota transplantation (FMT) has been shown to be effective in lowering the risk of diarrhea in piglets (Bin et al., 2018; Niederwerder et al., 2018). The mechanisms by which FMT decreases diarrhea in piglets remain to be determined, but they are probably multifaceted, such as enhanced beneficial microbes, increased microbiome diversity, and restored normal microbiota (Niederwerder, 2018) and maintenance of gut barrier functions (Cheng et al., 2018; Geng et al., 2018). No study has evaluated if FMT would affect calf diarrhea, which is more prevalent in dairy calves than in beef calves because dairy calves are separated from their mother soon after birth. The rumen microbiota and the fecal microbiota share many of the bacteria (Mao et al., 2015; Ozbayram et al., 2018), but the rumen microbiota has no or much less of the common enteric pathogens that cause calf diarrhea, such as enterotoxigenic *E. coli*, *Cryptosporidium parvum*, *Clostridium perfringens*, rotavirus, and coronavirus. Rumen microbiota can also be readily preserved in large quantities by lyophilization to facilitate repeated inoculation (Yu et al., 2020a). Therefore, rumen microbiota transplantation (RMT) may potentially be an effective strategy to prevent and decrease calf diarrhea in cattle herds, but future research using both FMT and RMT is warranted to confirm the utility of FMT and RMT in lowering calf diarrhea.

## Conclusion

Oral inoculation of pre-weaned young calves with rumen microbiota from adult cows may not substantially affect the overall rumen microbiota or its functional profiles but can affect the colonization of some rumen bacteria, methanogens, and protozoa and some metabolic pathways. Oral inoculation may also reduce the variable rumen microbiome among calves by decreasing the stochasticity of the rumen microbiota development. Increasing fiber content in starter feed or feeding of forage may be needed to maximize the effect of oral inoculation. In addition to reducing calf diarrhea, oral inoculation of young calves may also have other health benefits.

## Data Availability Statement

The datasets generated for this study can be found in the NCBI sequence read archive, accession number PRJNA562303.

## Ethics Statement

The animal study was reviewed and approved by Animal Care and Use Committee of the Institute of Animal Science, Chinese Academy of Agricultural Sciences.

## Author Contributions

DB and ZY conceived and designed the study. XZ and LM conducted the experiment and analyzed the animal data. TP and LW did the metataxonomic analysis. DB and ZY drafted the manuscript. TP, JX, and MW contributed to the writing of the manuscript. All the authors revised and edited the manuscript.

## Conflict of Interest

The authors declare that the research was conducted in the absence of any commercial or financial relationships that could be construed as a potential conflict of interest.

## Footnotes

1 https://huttenhower.sph.harvard.edu/galaxy/
