# Supplementary material for: Repeated Inoculation of Young Calves With Rumen Microbiota Does Not Significantly Modulate the Rumen Prokaryotic Microbiota Consistently but Decreases Diarrhea
Source: Front Microbiol. 2020 Jun 24;11:1403. doi: 10.3389/fmicb.2020.01403 (PMC7326819; doi:10.3389/fmicb.2020.01403)
Supplement: Supplementary file 1 [file Data_Sheet_1.docx]

Supplementary Table S1. Sequence data statistics.

|  | Donor* | Treatment* | | | | | SEM^⁑^ |
| --- | --- | --- | --- | --- | --- | --- | --- |
|  |  | Ctrl | |  | Inoc | |  |
|  |  | Ctrl-A | Ctrl-B |  | Inoc-A | Inoc-B |  |
| No. of input paired reads | 26,359 | 94,010 | 132,167 |  | 181,671 | 108,743 | 14,848 |
| Quality-filtered reads | 22,035 | 88,180 | 126,510 |  | 174,399 | 102,836 | 14,625 |
| Denoised reads | 19,498 | 87,322 | 125,904 |  | 173,658 | 102,111 | 14,596 |
| Merged reads | 10,722 | 80,957 | 119,539 |  | 165,229 | 96,085 | 14,087 |
| Chimera-filtered sequences | 8,660 | 64,305 | 101,902 |  | 144,674 | 82,935 | 12,602 |
| Taxa-filtered sequences** | 8,652 | 64,274 | 101,846 |  | 144,569 | 82,863 | 12,591 |

* Ctrl, control calves receiving autoclaved rumen fluid; Inoc, calves receiving fresh rumen fluid.

^⁑^ standard error of mean.

** Possible sequences of mitochondria and chloroplast were filtered out. Sequences that could not be assigned to Bacteria or Archaea were also excluded.

Supplementary Table S2. Comparison of OTUs* between the donors and their recipients and among the calf groups⁑

| OUT ID | Lowest taxon^⁂^ | Donor A (n=1) | Inoc-A (n=5) | Donor B (n=1) | Inoc-B (n=5) | Ctrl-A (n=4) | Ctrl-B (n=5) |
| --- | --- | --- | --- | --- | --- | --- | --- |
| 1 | p_SR1 | 0.299 |  | 0.233 | 0.001(2) |  |  |
| 2 | p_SR1 | 0.349 |  | 0.175 |  |  |  |
| 3 | o_*Bacteroidales* | 0.066 |  | 0.102 |  |  |  |
| 4 | o_*Bacteroidales* | 0.008 | 0.014 (5) |  | 0.107 (4) | 0.001 (1) | 0.001 (1) |
| 5 | o_*Bacteroidales* |  |  |  |  |  |  |
| 6 | o_*Bacteroidales* | 0.050 |  | 0.124 |  |  |  |
| 7 | o_*Bacteroidales* |  | 0.002 (3) |  | 0.041 (3) | 0.152 (2) | 0.310 (4) |
| 8 | o_*Bacteroidales* | 0.141 |  | 0.204 |  |  |  |
| 9 | o_*Bacteroidales* | 0.141 |  | 0.087 |  |  |  |
| 10 | o_*Bacteroidales* | 0.133 | 0.015 (5) | 0.146 | 0.009 (4) | 0.094 (3) | 0.002 (2) |
| 11 | o_*Bacteroidales* | 0.100 |  | 0.036 |  |  |  |
| 12 | o_*Bacteroidales* | 0.124 | 0.001 (3) | 0.204 | 0.001 (1) | 0.001 (1) | 0.002 (2) |
| 13 | o_*Bacteroidales* | 0.042 | 0.001 (2) | 0.226 | 0.003 (3) | 0.005 (3) |  |
| 14 | o_*Bacteroidales* | 0.141 | 0.006 (4) | 0.663 | 0.009 (4) | 0.023 (4) | 0.001 (3) |
| 15 | o_*Bacteroidales* | 0.133 |  | 0.138 |  |  |  |
| 16 | o_*Bacteroidales* | 1.088 |  | 2.040 |  |  |  |
| 17 | o_*Bacteroidales* | 0.216 | 0.001 (1) | 0.116 | 0.002 (2) | 0.003 (2) |  |
| 18 | o_*Bacteroidales* | 0.050 |  | 0.131 |  |  |  |
| 19 | o_*Bacteroidales* | 0.025 |  | 0.124 |  |  |  |
| 20 | o_Bacteroidales | 0.075 | 0.001 (1) | 0.124 |  |  |  |
| 21 | o_*Bacteroidales* | 0.365 |  | 0.058 |  |  |  |
| 22 | o_*Bacteroidales* | 0.050 | 0.001 (1) | 0.065 |  |  |  |
| 23 | o_*Bacteroidales* | 0.100 |  | 0.364 |  |  |  |
| 24 | o_*Bacteroidales* | 0.224 | 0.001 (4) | 0.357 | 0.093(5) | 0.068 (4) | 0.007 (5) |
| 25 | o_*Bacteroidales* | 0.399 | 0.001 (1) | 0.131 | 0.005(3) | 0.001 (1) |  |
| 26 | o_*Bacteroidales* | 0.465 |  | 0.452 |  |  |  |
| 27 | o_*Bacteroidales* | 0.075 |  | 0.218 | 0.001 (1) |  |  |
| 28 | o_*Bacteroidales* | 0.042 |  | 0.189 |  |  |  |
| 29 | o_*Bacteroidales* | 0.266 | 0.001 (2) | 0.306 | 0.002 (2) | 0.002 (2) | 0.001 (1) |
| 30 | o_*Bacteroidales* |  | 0.008 (3) | 0.036 | 0.047 (4) | 0.008 (3) | 0.003 (1) |
| 31 | o_*Bacteroidales* | 0.033 |  | 0.102 |  |  |  |
| 32 | o_*Bacteroidales* | 0.149 |  | 0.124 |  |  |  |
| 33 | o_*Clostridiales* |  |  |  | 0.030 (1) | 0.003 (2) |  |
| 34 | o_*Clostridiales* |  |  |  | 0.030 (2) |  |  |
| 35 | o_*Clostridiales* |  | 0.053 (3) |  | 0.196 (4) | 0.002 (1) | 0.001 (2) |
| 36 | o_*Clostridiales* |  | 0.068 (4) |  | 0.030 (4) | 0.045 (3) | 0.020 (3) |
| 37 | o_*Clostridiales* | 0.025 |  | 0.233 |  |  |  |
| 38 | o_*Clostridiales* |  | 0.034 (4) |  | 0.001 (2) |  | 0.010 (3) |
| 39 | o_*Clostridiales* |  | 0.221 (5) |  | 0.010 (3) | 0.004 (3) | 0.044 (4) |
| 40 | o_*Clostridiales* | 0.100 |  | 0.029 |  | 0.035 (1) |  |
| 41 | o_*Clostridiales* |  | 0.103 (5) | 0.007 | 0.447 (5) | 0.472 (4) | 0.011 (4) |
| 42 | o_*Clostridiales* |  | 0.001 (3) |  | 0.047 (2) | 0.002 (2) | 0.001 (2) |
| 43 | o_*Clostridiales* |  | 0.002 (5) |  | 0.050 (4) | 0.001 (1) | 0.002 (4) |
| 44 | o_*Clostridiales* | 0.124 | 0.001 (2) | 0.109 | 0.001 (2) |  | 0.002 (3) |
| 45 | o_*Clostridiales* |  | 0.177 (5) |  | 0.510 (5) | 0.378 (4) | 0.078 (5) |
| 46 | o_*Clostridiales* | 0.174 | 0.001 (1) | 0.182 |  |  |  |
| 47 | o_*Clostridiales* | 0.108 |  |  |  |  |  |
| 48 | o_*Clostridiales* | 0.050 |  | 0.124 |  |  |  |
| 49 | o_*Clostridiales* | 0.075 |  | 0.291 | 0.008 (1) |  |  |
| 50 | o_*Clostridiales* |  |  | 0.175 |  |  |  |
| 51 | o_*Clostridiales* | 0.124 |  | 0.175 |  |  |  |
| 52 | o_*Clostridiales* |  | 0.011 (5) |  | 0.124 (4) | 0.019 (2) | 0.006 (4) |
| 53 | o_*Clostridiales* | 0.033 | 0.007 (5) | 0.029 | 0.037 (2) | 0.006 (3) | 0.003 (4) |
| 54 | o_*Clostridiales* |  | 0.147 (5) |  | 0.222 (5) | 0.191 (4) | 0.079 (4) |
| 55 | o_RF39 |  | 0.014 (5) |  | 0.004 (4) | 0.052 (4) | 0.010 (4) |
| 56 | o_YS2 | 0.008 | 0.048 (5) |  | 0.011 (3) | 0.036 (3) | 0.036 (3) |
| 57 | f_[*Mogibacteriaceae*] |  | 0.001 (1) |  | 0.035 (2) | 0.001 (1) |  |
| 58 | f_[*Mogibacteriaceae*] |  | 0.446 (5) |  | 0.659 (5) | 0.127 (4) | 0.036 (5) |
| 59 | f_[*Mogibacteriaceae*] |  | 0.026 (4) |  | 0.046 (4) | 0.107 (3) | 0.004 (3) |
| 60 | f_[*Mogibacteriaceae*] |  | 0.001 (2) |  | 0.048 (3) | 0.001 (1) |  |
| 61 | f_[*Mogibacteriaceae*] |  | 0.004 (5) |  | 0.031 (4) | 0.012 (2) | 0.003 (2) |
| 62 | f_[*Paraprevotellaceae*] | 0.158 |  | 0.131 |  |  |  |
| 63 | f_[*Paraprevotellaceae*] | 0.008 |  | 0.109 |  |  |  |
| 64 | f_[*Paraprevotellaceae*] | 0.789 | 0.001 (1) | 0.714 |  | 0.046 (1) |  |
| 65 | f_[*Paraprevotellaceae*] | 0.124 | 0.001 (1) | 0.066 | 0.001 (2) | 0.003 (1) | 0.001 (2) |
| 66 | f_[*Paraprevotellaceae*] | 0.058 |  | 0.109 |  |  |  |
| 67 | f_[*Paraprevotellaceae*] |  | 0.028 (4) |  | 0.012 (4) | 0.128 (3) | 0.012 (4) |
| 68 | f_BS11 | 0.133 |  | 0.066 | 0.001 (1) |  |  |
| 69 | f_BS11 | 0.440 |  | 0.364 | 0.001 (1) | 0.001 (1) |  |
| 70 | f_BS11 | 0.440 |  | 0.138 |  |  |  |
| 71 | f_*Christensenellaceae* | 0.166 |  | 0.044 |  |  |  |
| 72 | f_*Christensenellaceae* | 0.124 |  | 0.124 | 0.001 (1) |  |  |
| 73 | f_*Coriobacteriaceae* |  | 0.001 (2) |  | 0.054 (2) | 0.002 (1) |  |
| 74 | f_*Coriobacteriaceae* |  | 0.001 (3) | 0.007 | 0.034 (3) |  |  |
| 75 | f_*Coriobacteriaceae* |  | 0.002 (3) |  | 0.042 (5) | 0.007 (2) | 0.003 (4) |
| 76 | f_*Coriobacteriaceae* |  | 0.003 (3) |  | 0.225 (4) | 0.013 (2) | 0.006 (4) |
| 77 | f_*Coriobacteriaceae* |  | 0.001 (4) |  | 0.060 (4) | 0.008 (3) | 0.002 (3) |
| 78 | f_*Coriobacteriaceae* | 0.008 | 0.010 (5) |  | 0.233 (5) | 0.043 (4) | 0.003 (4) |
| 79 | f_*Coriobacteriaceae* |  | 0.004 (5) |  | 0.064 (3) | 0.023 (4) | 0.003 (5) |
| 80 | f_*Coriobacteriaceae* |  | 0.001 (2) |  | 0.022 (3) | 0.001 (1) | 0.001 (1) |
| 81 | f_*Coriobacteriaceae* |  | 0.034 (5) |  | 1.250 (5) | 0.047 (4) | 0.025 (5) |
| 82 | f_*Lachnospiraceae* |  | 0.001 (1) |  | 0.026 (2) |  |  |
| 83 | f_*Lachnospiraceae* |  | 0.001 (2) |  | 0.052 (3) |  |  |
| 84 | f_*Lachnospiraceae* |  | 0.023 (3) |  | 0.001 (1) | 0.023 (1) | 0.001 (4) |
| 85 | f_*Lachnospiraceae* |  | 0.021 (3) |  | 0.004 (2) | 0.036 (4) | 0.003 (4) |
| 86 | f_*Lachnospiraceae* |  | 0.005 (3) |  | 0.001 (1) | 0.034 (2) | 0.006 (3) |
| 87 | f_*Lachnospiraceae* |  | 0.045 (3) |  | 0.021 (4) | 0.046 (3) | 0.001 (3) |
| 88 | f_*Lachnospiraceae* |  | 0.010 (3) |  | 0.001 (1) | 0.055 (2) | 0.011 (3) |
| 89 | f_*Lachnospiraceae* |  | 0.038 (4) |  | 0.004 (3) | 0.015 (3) | 0.009 (3) |
| 90 | f_*Lachnospiraceae* |  | 0.066 (5) |  | 0.167 (5) | 0.089 (3) | 0.024 (4) |
| 91 | f_*Lachnospiraceae* |  |  |  | 0.044 (2) | 0.001 (2) |  |
| 92 | f_*Lachnospiraceae* |  | 0.011 (3) |  | 0.004 (4) | 0.078 (3) | 0.003 (4) |
| 93 | f_*Lachnospiraceae* |  | 0.037 (5) |  | 0.185 (5) | 0.059 (4) | 0.036 (5) |
| 94 | f_*Lachnospiraceae* | 0.033 | 0.006 (4) | 0.058 | 0.376 (3) | 0.055 (2) | 0.002 (3) |
| 95 | f_*Lachnospiraceae* |  | 0.039 (5) |  | 0.195 (5) | 0.001 (1) | 0.012 (4) |
| 96 | f_*Lachnospiraceae* |  | 0.057 (5) |  | 0.085 (4) | 0.040 (4) | 0.020 (5) |
| 97 | f_*Lachnospiraceae* | 0.124 |  | 0.073 |  |  |  |
| 98 | f_*Lachnospiraceae* |  | 0.012 (5) |  | 0.039 (5) | 0.118 (4) | 0.007 (5) |
| 99 | f_*Lachnospiraceae* |  | 0.147 (5) |  | 0.065 (5) | 0.181 (4) | 0.183 (5) |
| 100 | f_*Lachnospiraceae* |  | 0.017 (3) |  | 0.498 (3) |  | 0.001 (1) |
| 101 | f_*Lachnospiraceae* |  | 0.007 (5) |  | 0.035 (5) | 0.014 (4) | 0.003 (4) |
| 102 | f_*Lachnospiraceae* |  | 0.342 (5) | 0.073 | 0.186 (5) | 0.292 (4) | 0.047 (5) |
| 103 | f_*Lachnospiraceae* |  | 0.001 (3) |  | 0.004 (3) | 0.191 (3) | 0.002 (4) |
| 105 | f_RF16 | 0.639 |  | 0.168 |  |  |  |
| 106 | f_RF16 | 1.478 |  | 1.450 | 0.001 (1) |  |  |
| 107 | f_RF16 | 0.133 |  | 0.014 |  |  |  |
| 108 | f_*Ruminococcaceae* |  |  |  | 0.046 (2) |  |  |
| 109 | f_*Ruminococcaceae* |  | 0.041 (5) |  | 0.054 (5) | 0.137 (4) | 0.012 (4) |
| 110 | f_*Ruminococcaceae* |  | 0.033 (3) |  |  | 0.009 (1) | 0.001 (1) |
| 111 | f_*Ruminococcaceae* |  |  |  | 0.043 (1) |  |  |
| 112 | f_*Ruminococcaceae* |  |  |  | 0.046 (3) |  |  |
| 113 | f_*Ruminococcaceae* |  | 0.013 (4) |  | 8.631 (5) | 0.003 (1) | 0.001 (2) |
| 114 | f_*Ruminococcaceae* |  | 0.001 (1) |  | 0.066 (5) | 0.001 (1) |  |
| 115 | f_*Ruminococcaceae* |  | 0.030 (4) |  | 0.691 (5) | 0.020 (3) | 0.012 (4) |
| 116 | f_*Ruminococcaceae* |  |  |  |  |  |  |
| 117 | f_*Ruminococcaceae* | 0.191 | 0.001 (2) | 0.270 | 0.001 (2) | 0.001 (1) |  |
| 118 | f_*Ruminococcaceae* |  | 0.034 (4) |  | 0.369 (5) | 0.035 (3) | 0.029 (4) |
| 119 | f_*Ruminococcaceae* | 0.133 | 0.001 (1) | 0.168 |  | 0.001 (1) |  |
| 120 | f_*Ruminococcaceae* | 0.017 |  | 0.051 | 0.030 (1) |  |  |
| 121 | f_*Ruminococcaceae* | 0.133 | 0.001 (1) | 0.051 | 0.001(1) |  |  |
| 122 | f_*Ruminococcaceae* | 0.066 |  | 0.401 |  |  |  |
| 123 | f_*Ruminococcaceae* |  | 0.015 (2) |  | 0.507 (2) | 0.037 (2) | 0.001 (1) |
| 124 | f_*Ruminococcaceae* | 0.124 | 0.001 (1) | 0.226 | 0.001 (1) | 0.001 (1) |  |
| 125 | f_*Ruminococcaceae* |  | 0.090 (4) |  | 0.033 (4) | 0.120 (3) | 0.001 (1) |
| 126 | f_Ruminococcaceae | 0.141 | 0.005 (4) | 0.095 | 0.002 (3) | 0.002 (2) | 0.001 (2) |
| 127 | f_*Ruminococcaceae* | 0.241 | 0.001 (3) | 0.372 | 0.004 (3) | 0.001 (2) | 0.001 (2) |
| 128 | f_S24-7 | 0.050 | 0.001 (1) | 0.168 | 0.001 (1) |  |  |
| 129 | f_S24-7 |  | 0.004 (3) |  | 0.057 (2) | 0.009 (3) | 0.021 (5) |
| 130 | f_S24-7 | 0.017 |  | 0.044 | 0.150 (4) | 0.011 (1) |  |
| 131 | f_S24-7 |  |  | 0.102 |  |  |  |
| 132 | f_S24-7 | 0.066 |  | 0.568 |  |  |  |
| 133 | f_S24-7 | 0.042 |  | 0.102 |  |  |  |
| 134 | f_S24-7 | 0.083 |  | 0.218 | 0.001 (1) |  |  |
| 135 | f_S24-7 |  | 0.020 (4) |  | 0.177 (4) | 0.055 (4) | 0.076 (5) |
| 136 | f_S24-7 |  |  | 0.036 |  |  |  |
| 137 | f_S24-7 | 0.058 |  | 0.175 | 0.001 (1) |  |  |
| 138 | f_S24-7 | 0.017 |  | 0.189 |  |  |  |
| 139 | f_S24-7 | 0.066 | 0.001 (2) | 0.393 | 0.008 (3) | 0.004 (1) |  |
| 140 | f_S24-7 | 0.316 |  | 0.510 |  |  |  |
| 141 | f_S24-7 | 0.149 |  | 0.335 |  |  |  |
| 142 | f_S24-7 | 0.133 |  | 0.058 |  |  |  |
| 143 | f_S24-7 | 0.017 |  | 0.124 |  |  |  |
| 144 | f_S24-7 | 0.216 |  | 0.320 |  |  |  |
| 145 | f_S24-7 | 0.066 |  | 0.131 |  |  |  |
| 146 | f_S24-7 |  | 0.154 (5) |  | 1.127 (5) | 0.280 (4) | 0.480 (5) |
| 147 | f_S24-7 |  | 0.017 (5) |  | 0.205 (4) | 0.054 (4) | 0.090 (5) |
| 148 | f_S24-7 | 0.066 | 0.001 (2) | 0.036 | 0.004 (4) | 0.016 (2) |  |
| 149 | f_S24-7 | 0.108 |  | 0.153 | 0.001 (1) |  |  |
| 150 | f_S24-7 | 0.166 |  | 0.459 |  |  |  |
| 151 | f_S24-7 | 0.058 |  | 0.189 |  |  |  |
| 152 | f_*Succinivibrionaceae* |  |  | 0.007 |  | 0.001 (1) |  |
| 153 | f_*Succinivibrionaceae* |  | 0.001 (1) | 0.022 |  |  | 0.001 (1) |
| 154 | f_*Succinivibrionaceae* |  | 0.044 (5) | 0.014 | 0.030 (4) | 0.035 (4) | 0.084 (5) |
| 155 | f_*Succinivibrionaceae* |  | 0.030 (5) | 0.022 | 0.057 (3) | 0.022 (4) | 0.092 (5) |
| 156 | f_*Succinivibrionaceae* | 0.141 |  | 0.066 |  |  |  |
| 157 | f_*Succinivibrionaceae* |  | 0.093 (5) | 0.014 | 0.099 (4) | 0.070 (4) | 0.076 (5) |
| 158 | f_*Succinivibrionaceae* | 0.124 |  | 0.029 |  |  |  |
| 159 | f_*Succinivibrionaceae* |  | 0.099 (5) | 0.022 | 0.080 (4) | 0.057 (4) | 0.068 (5) |
| 160 | f_*Succinivibrionaceae* | 0.390 |  | 0.102 |  |  |  |
| 161 | f_*Succinivibrionaceae* | 0.025 | 41.217 (5) | 1.952 | 22.229 (5) | 3.409 (4) | 39.675 (5) |
| 162 | f_*Succinivibrionaceae* | 0.523 |  | 0.080 |  |  |  |
| 163 | f_*Succinivibrionaceae* | 10.746 |  | 4.058 |  |  |  |
| 164 | f_Veillonellaceae |  | 0.015 (5) |  | 0.006 (3) | 0.060 (3) | 0.009 (4) |
| 165 | f_*Veillonellaceae* |  | 0.011 (5) |  | 0.010 (3) | 0.092 (3) | 0.043 (4) |
| 166 | f_*Veillonellaceae* |  | 0.014 (5) |  | 0.003 (2) | 0.034 (4) | 0.050 (4) |
| 167 | f_*Veillonellaceae* |  | 0.022 (4) | 0.007 | 0.002 (3) | 0.127 (2) | 0.008 (3) |
| 168 | f_*Veillonellaceae* |  | 0.010 (4) |  | 0.002 (3) | 0.099 (3) | 0.021 (5) |
| 169 | f_*Veillonellaceae* |  | 0.016 (4) |  | 0.005 (2) | 0.169 (2) | 0.036 (4) |
| 170 | f_*Veillonellaceae* |  | 0.006 (3) |  | 0.006 (1) | 0.006 (2) | 0.037 (3) |
| 171 | f_*Veillonellaceae* |  | 0.005 (5) |  | 0.002 (3) | 0.052 (4) | 0.005 (4) |
| 172 | f_*Veillonellaceae* |  | 0.122 (4) | 0.007 | 0.038 (5) | 0.019 (4) | 0.033 (4) |
| 173 | f_*Veillonellaceae* |  | 0.062 (5) |  | 0.021 (5) | 0.221 (4) | 0.040 (4) |
| 174 | f_*Veillonellaceae* | 0.033 |  | 0.102 |  | 0.001 (1) |  |
| 175 | f_*Veillonellaceae* |  | 0.038 (5) |  | 0.004 (3) | 0.285 (4) | 0.010 (4) |
| 176 | f_*Veillonellaceae* |  | 0.003 (3) |  | 0.001 (1) | 0.028 (1) | 0.004 (2) |
| 177 | f_*Veillonellaceae* |  | 0.333 (5) |  | 0.053 (5) | 1.181 (4) | 0.184 (5) |
| 178 | f_*Veillonellaceae* |  | 0.300 (4) |  | 0.131 (4) | 1.115 (2) | 1.464 (5) |
| 179 | f_*Veillonellaceae* |  | 0.016 (5) |  | 0.107 (4) | 0.064 (4) | 0.024 (5) |
| 180 | f_*Veillonellaceae* |  | 0.013 (5) |  | 0.044 (3) | 0.039 (4) | 0.015 (5) |
| 181 | g_[*Prevotella*] |  | 0.007 (1) |  | 0.002 (2) | 0.063 (2) | 0.001 (1) |
| 182 | g_*Acidaminococcus* |  | 0.055 (5) |  | 0.043 (5) | 0.106 (4) | 0.174 (5) |
| 183 | g_*Acidaminococcus* |  | 0.215 (5) |  | 0.059 (5) | 0.378 (4) | 0.292 (5) |
| 184 | g_*Anaeroplasma* | 0.033 |  | 0.015 |  |  |  |
| 185 | g_*Anaerovibrio* | 0.058 | 0.001 (3) | 0.146 |  | 0.001 (2) |  |
| 186 | g_BF311 | 0.149 |  |  |  | 0.001 (1) |  |
| 187 | g_*Bifidobacterium* |  | 0.001 (1) |  |  | 0.133 (2) | 0.176 (1) |
| 188 | g_*Bifidobacterium* |  | 0.311 (4) |  | 0.474 (5) | 1.682 (4) | 0.295 (4) |
| 189 | g_*Bifidobacterium* |  | 0.003 (3) |  | 0.014 (3) | 0.084 (4) | 0.003 (3) |
| 190 | g_*Bulleidia* |  | 0.015 (2) |  | 1.361 (4) | 0.004 (1) | 0.002 (1) |
| 191 | g_*Butyrivibrio* |  | 0.012 (2) |  | 0.146 (4) | 0.020 (3) | 0.001 (1) |
| 192 | g_*Butyrivibrio* |  | 0.008 (4) |  | 0.049 (5) | 0.010 (2) | 0.001 (3) |
| 193 | g_*Butyrivibrio* |  | 0.008 (5) |  | 0.156 (4) | 0.029 (3) | 0.003 (3) |
| 194 | g_*Butyrivibrio* | 0.017 |  | 0.357 |  |  |  |
| 195 | g_*Butyrivibrio* | 0.075 |  | 0.262 |  |  |  |
| 196 | g_*Butyrivibrio* |  | 0.222 (5) |  | 1.928 (5) | 0.078 (4) | 0.019 (5) |
| 197 | g_*Butyrivibrio* | 0.008 | 0.001 (2) |  | 0.061 (2) | 0.001 (1) |  |
| 198 | g_*Butyrivibrio* |  | 0.022 (5) |  | 0.060 (4) | 1.981 (4) | 0.016 (4) |
| 199 | g_*Butyrivibrio* | 0.216 |  | 0.248 |  |  |  |
| 200 | g_*Butyrivibrio* |  | 0.095 (4) | 0.007 | 0.213 (3) | 0.014 (4) | 0.003 (4) |
| 201 | g_*Catenibacterium* |  | 0.001 (2) | 0.051 |  | 0.001 (1) |  |
| 202 | g_CF231 | 0.291 |  | 0.124 |  |  |  |
| 203 | g_CF231 | 0.108 |  | 0.146 |  |  |  |
| 204 | g_CF231 | 0.058 |  | 0.102 |  |  |  |
| 205 | g_CF231 | 0.191 |  | 0.197 |  |  |  |
| 206 | g_*Clostridium* |  | 0.024 (3) |  | 0.059 (3) | 0.125 (4) | 0.009 (3) |
| 207 | g_*Clostridium* |  | 0.020 (4) |  | 0.023 (3) | 0.057 (4) | 0.007 (1) |
| 208 | g_*Clostridium* |  | 0.054 (5) |  | 0.083 (4) | 0.090 (4) | 0.044 (5) |
| 209 | g_*Coprococcus* |  | 0.008 (5) |  | 0.222 (5) | 0.007 (3) | 0.015 (4) |
| 210 | g_*Coprococcus* | 0.100 |  | 0.124 | 0.001 (1) | 0.001 (1) |  |
| 211 | g_*Desulfovibrio* |  | 0.002 (3) | 0.014 | 0.024 (3) | 0.005 (2) | 0.002 (3) |
| 212 | g_*Desulfovibrio* |  | 0.092 (5) |  | 0.103 (5) | 0.282 (4) | 0.113 (5) |
| 213 | g_*Dialister* |  | 0.017 (3) |  | 0.003 (2) | 0.014 (1) | 0.025 (3) |
| 214 | g_*Dialister* |  | 0.015 (4) |  | 0.004 (2) | 0.042 (3) | 0.006 (3) |
| 215 | g_*Dialister* |  | 0.732 (5) |  | 0.495 (4) | 1.830 (4) | 0.479 (5) |
| 216 | g_L7A_E11 |  |  |  | 0.043 (1) |  | 0.001 (1) |
| 217 | g_*Megasphaera* |  | 0.085 (5) |  | 0.064 (4) | 0.234 (4) | 0.056 (5) |
| 218 | g_*Megasphaera* |  | 0.050 (5) |  | 0.001 (2) | 0.716 (4) | 0.132 (5) |
| 219 | g_*Methanobrevibacter* |  | 0.006 (2) |  | 0.069 (4) | 0.070 (4) | 0.006 (3) |
| 220 | g_*Methanobrevibacter* |  | 0.028 (2) | 0.277 | 0.001 (2) |  |  |
| 221 | g_*Mitsuokella* |  | 0.020 (5) |  | 0.007 (5) | 0.030 (4) | 0.006 (4) |
| 222 | g_*Mitsuokella* |  | 0.246 (5) | 0.007 | 0.074 (5) | 0.376 (4) | 0.148 (5) |
| 223 | g_*Mogibacterium* |  |  |  | 0.031 (1) | 0.001 (1) |  |
| 224 | g_*Mogibacterium* |  | 0.014 (5) |  | 0.520 (4) | 0.006 (2) | 0.004 (3) |
| 225 | g_*Oscillospira* |  | 0.022 (5) | 0.014 | 0.165 (5) | 0.036 (4) | 0.008 (5) |
| 226 | g_*Prevotella* | 0.075 |  | 0.087 |  |  |  |
| 227 | g_*Prevotella* |  | 0.013 (4) |  | 0.009 (3) | 0.074 (4) | 0.014 (4) |
| 228 | g_*Prevotella* | 0.141 |  | 0.044 |  |  |  |
| 229 | g_*Prevotella* |  | 0.003 (4) |  | 0.025 (3) | 0.082 (3) | 0.034 (5) |
| 230 | g_*Prevotella* | 0.058 |  |  |  |  |  |
| 231 | g_*Prevotella* |  | 0.461 (5) |  | 0.460 (5) | 1.133 (4) | 0.125 (5) |
| 232 | g_*Prevotella* | 0.158 |  | 0.073 |  |  |  |
| 233 | g_*Prevotella* |  | 0.010 (4) |  | 0.024 (4) | 0.033 (3) | 0.047 (4) |
| 234 | f_Ruminococcaceae |  | 0.002 (2) |  | 0.004 (3) | 0.053 (2) | 0.021 (5) |
| 235 | g_*Prevotella* |  | 0.001 (1) |  | 0.025 (4) | 0.003 (2) | 0.001 (1) |
| 236 | g_*Prevotella* |  | 0.024 (4) |  | 0.013 (4) | 0.105 (3) | 0.097 (5) |
| 237 | g_*Prevotella* | 0.116 | 0.001 (2) | 0.080 | 0.024 (3) | 0.046 (1) |  |
| 238 | g_*Prevotella* | 0.116 |  | 0.051 |  |  |  |
| 239 | g_*Prevotella* | 0.025 |  | 0.102 |  |  |  |
| 240 | g_*Prevotella* |  | 0.012 (4) |  | 0.039 (3) | 0.060 (4) | 0.014 (3) |
| 241 | g_*Prevotella* |  | 0.033 (5) |  | 0.007 (2) | 0.058 (3) | 0.033 (3) |
| 242 | g_*Prevotella* |  | 0.659 (5) |  | 0.740 (4) | 1.008 (4) | 0.409 (5) |
| 243 | g_*Prevotella* |  | 0.014 (5) |  | 0.027 (3) | 0.032 (3) | 0.005 (4) |
| 244 | g_*Prevotella* |  | 0.002 (3) |  | 0.018 (2) | 0.037 (3) | 0.026 (4) |
| 245 | g_*Prevotella* | 0.017 |  | 0.124 |  |  | 0.001 (1) |
| 246 | g_*Prevotella* |  | 0.012 (4) |  | 0.028 (3) | 0.002 (1) | 0.020 (3) |
| 247 | g_*Prevotella* |  | 0.005 (3) |  | 0.051 (3) | 0.020 (3) | 0.043 (4) |
| 248 | g_*Prevotella* |  | 0.021 (5) |  | 0.004 (3) | 0.048 (3) | 0.031 (4) |
| 249 | g_*Prevotella* | 0.158 |  | 0.131 |  |  |  |
| 250 | g_*Prevotella* | 0.174 |  | 0.124 |  |  |  |
| 251 | g_*Prevotella* |  | 0.027 (3) |  | 0.073 (3) | 0.043 (3) | 0.001 (2) |
| 252 | g_*Prevotella* | 0.183 |  | 0.124 |  |  |  |
| 253 | g_*Prevotella* |  | 0.294 (5) |  | 0.315 (4) | 0.410 (4) | 0.247 (5) |
| 254 | g_*Prevotella* |  |  | 0.029 |  |  |  |
| 255 | g_*Prevotella* | 0.108 |  | 0.029 |  |  |  |
| 256 | g_*Prevotella* |  |  | 0.102 |  |  |  |
| 257 | g_*Prevotella* |  | 0.290 (5) |  | 0.390 (4) | 0.534 (4) | 0.204 (4) |
| 258 | g_*Prevotella* |  | 0.029 (5) |  | 0.006 (3) | 0.013 (2) | 0.023 (5) |
| 259 | g_*Prevotella* |  | 0.011 (5) |  | 0.039 (4) | 0.046 (4) | 0.008 (4) |
| 260 | g_*Prevotella* |  | 0.024 (4) |  | 0.060 (4) | 0.039 (3) | 0.001 (2) |
| 261 | g_*Prevotella* |  | 0.004 (2) |  | 0.089 (2) | 0.011 (4) | 0.018 (3) |
| 262 | g_*Prevotella* | 0.033 |  | 0.073 |  |  |  |
| 263 | g_*Prevotella* | 0.042 |  | 0.153 | 0.001 (1) |  |  |
| 264 | g_*Prevotella* | 0.083 |  | 0.029 |  |  |  |
| 265 | g_*Prevotella* |  | 0.010 (5) |  | 0.044 (3) | 0.014 (4) | 0.020 (4) |
| 266 | g_*Prevotella* |  | 0.045 (5) |  | 0.023 (5) | 0.075 (4) | 0.024 (5) |
| 267 | g_*Prevotella* | 0.141 |  | 0.197 |  |  |  |
| 268 | g_*Prevotella* |  | 0.011 (4) |  | 0.022 (4) | 0.048 (4) | 0.061 (5) |
| 269 | g_*Prevotella* |  | 0.016 (4) |  | 0.090 (4) | 0.109 (4) | 0.031 (5) |
| 270 | g_*Prevotella* |  | 0.051 (5) |  | 0.103 (4) | 0.042 (4) | 0.110 (5) |
| 271 | g_*Prevotella* |  | 0.046 (5) |  | 0.009 (4) | 0.052 (4) | 0.081 (5) |
| 272 | g_*Prevotella* | 0.149 |  | 0.218 |  |  |  |
| 273 | g_*Prevotella* |  | 0.051 (5) |  | 0.008 (3) | 0.017 (3) | 0.031 (4) |
| 274 | g_*Prevotella* |  | 0.008 (4) |  | 0.007 (4) | 0.050 (3) | 0.035 (5) |
| 275 | g_*Prevotella* |  | 0.024 (5) |  | 0.028 (4) | 0.054 (4) | 0.012 (5) |
| 276 | g_*Prevotella* |  | 0.051 (3) |  | 0.160 (3) |  |  |
| 277 | g_*Prevotella* |  | 0.017 (5) |  | 0.027 (3) | 0.020 (4) | 0.031 (5) |
| 278 | g_*Prevotella* |  |  | 0.007 |  |  |  |
| 279 | g_*Prevotella* | 0.124 |  | 0.029 |  | 0.001 (1) |  |
| 280 | g_*Prevotella* | 0.042 |  | 0.029 |  |  |  |
| 281 | g_*Prevotella* | 0.025 |  | 0.007 |  |  |  |
| 282 | g_*Prevotella* | 0.141 |  | 0.080 |  |  |  |
| 283 | g_*Prevotella* |  | 0.014 (4) |  | 0.080 (3) | 0.016 (4) | 0.043 (5) |
| 284 | g_*Prevotella* |  |  |  |  | 0.027 (1) |  |
| 285 | g_*Prevotella* |  | 0.111 (5) |  | 0.177 (4) | 0.430 (4) | 0.583 (5) |
| 286 | g_*Prevotella* | 0.075 |  | 0.109 |  |  |  |
| 287 | g_*Prevotella* |  | 0.006 (4) |  | 0.049 (3) | 0.018 (3) | 0.008 (4) |
| 288 | g_*Prevotella* |  | 0.002 (1) |  |  |  | 0.041 (1) |
| 289 | g_*Prevotella* | 0.008 |  | 0.022 |  |  |  |
| 290 | g_*Prevotella* |  | 0.014 (4) |  | 0.020 (4) | 0.169 (2) | 0.038 (5) |
| 291 | g_*Prevotella* |  | 0.017 (2) |  |  | 0.026 (1) | 0.001 (1) |
| 292 | g_*Prevotella* | 0.025 |  | 0.007 |  |  |  |
| 293 | g_*Prevotella* | 0.133 |  | 0.095 |  |  |  |
| 294 | g_*Prevotella* |  | 0.031 (5) |  | 0.007 (4) | 0.032 (4) | 0.039 (5) |
| 295 | g_*Prevotella* | 0.017 |  | 0.102 |  |  |  |
| 296 | g_*Prevotella* |  | 0.001 (1) |  |  | 0.063 (1) |  |
| 297 | g_*Prevotella* | 0.183 |  | 0.080 |  |  |  |
| 298 | g_*Prevotella* |  | 0.036 (5) |  | 0.124 (4) | 0.185 (4) | 0.012 (5) |
| 299 | g_*Prevotella* |  | 0.007 (5) |  | 0.006 (4) | 0.302 (4) | 0.054 (5) |
| 300 | g_*Prevotella* | 0.083 |  | 0.124 |  |  |  |
| 301 | g_*Prevotella* |  | 1.068 (5) |  | 1.779 (5) | 0.924 (4) | 1.228 (5) |
| 302 | g_*Prevotella* | 0.166 |  | 0.051 |  |  |  |
| 303 | g_*Prevotella* |  | 0.004 (4) |  | 0.029 (3) | 0.026 (3) | 0.008 (5) |
| 304 | g_*Prevotella* |  | 0.006 (4) |  | 0.147 (4) | 0.032 (2) | 0.032 (5) |
| 305 | g_*Prevotella* |  | 0.041 (5) |  | 0.065 (5) | 0.073 (4) | 0.027 (5) |
| 306 | g_*Prevotella* |  | 0.026 (5) |  | 0.050 (4) | 0.026 (4) | 0.062 (5) |
| 307 | g_*Prevotella* |  | 0.015 (5) |  | 0.035 (3) | 0.021 (4) | 0.034 (4) |
| 308 | g_*Prevotella* | 0.008 |  | 0.131 |  |  |  |
| 309 | g_*Prevotella* | 0.116 |  | 0.073 |  |  |  |
| 310 | g_*Prevotella* |  |  | 0.014 | 0.031 (2) |  |  |
| 311 | g_*Prevotella* |  | 0.021 (5) |  | 0.039 (5) | 0.085 (3) | 0.112 (5) |
| 312 | g_*Prevotella* |  | 0.235 (5) |  | 0.026 (3) | 0.334 (3) | 0.048 (5) |
| 313 | g_*Prevotella* |  | 0.029 (5) |  | 0.035 (3) | 0.075 (4) | 0.015 (4) |
| 314 | g_*Prevotella* |  | 0.024 (4) |  | 0.014 (3) | 0.030 (2) | 0.007 (3) |
| 315 | g_*Prevotella* |  | 0.056 (5) | 0.007 | 0.022 (4) | 0.042 (3) | 0.007 (3) |
| 316 | g_*Prevotella* | 0.141 |  | 0.153 |  |  |  |
| 317 | g_*Prevotella* | 0.075 |  | 0.153 |  |  |  |
| 318 | g_*Prevotella* | 0.448 |  | 0.233 |  |  |  |
| 319 | g_*Prevotella* |  | 0.017 (3) |  | 0.017 (3) | 0.032 (2) | 0.004 (3) |
| 320 | g_*Prevotella* |  |  | 0.073 |  |  |  |
| 321 | g_*Prevotella* | 0.149 |  | 0.131 |  |  |  |
| 322 | g_*Prevotella* |  | 0.017 (3) |  | 0.018 (3) | 0.036 (2) | 0.002 (1) |
| 323 | g_*Prevotella* |  | 0.008 (3) |  | 0.024 (3) | 0.023 (4) | 0.038 (4) |
| 324 | g_*Prevotella* |  | 0.070 (4) |  | 0.159 (4) | 0.337 (4) | 0.242 (5) |
| 325 | g_*Prevotella* |  |  | 0.299 |  |  |  |
| 326 | g_*Prevotella* |  | 0.139 (5) |  | 0.096 (5) | 0.168 (4) | 0.073 (5) |
| 327 | g_*Prevotella* |  | 0.064 (2) |  | 0.001 (2) | 0.019 (2) | 0.003 (1) |
| 328 | g_*Prevotella* | 0.166 |  | 0.080 |  |  |  |
| 329 | g_*Prevotella* | 0.116 |  | 0.058 |  |  |  |
| 330 | g_*Prevotella* | 0.058 |  | 0.102 |  |  |  |
| 331 | g_*Prevotella* | 0.208 |  | 0.073 |  |  |  |
| 332 | g_*Prevotella* |  | 0.019 (4) |  | 0.038 (3) | 0.060 (3) | 0.005 (4) |
| 333 | g_*Prevotella* |  | 0.379 (5) |  | 0.063 (5) | 0.608 (3) | 0.313 (5) |
| 334 | g_*Prevotella* |  | 0.073 (5) |  | 0.067 (3) | 0.148 (3) | 0.011 (4) |
| 335 | g_*Prevotella* |  | 0.014 (5) |  | 0.051 (4) | 0.036 (4) | 0.025 (4) |
| 336 | g_*Prevotella* |  | 0.010 (5) |  | 0.032 (3) | 0.036 (4) | 0.031 (5) |
| 337 | g_*Prevotella* |  | 0.053 (5) |  | 0.055 (4) | 0.082 (4) | 0.029 (4) |
| 338 | g_*Prevotella* |  | 0.021 (5) |  | 0.040 (2) | 0.030 (4) | 0.003 (2) |
| 339 | g_*Prevotella* |  | 0.014 (5) |  | 0.010 (3) | 0.036 (4) | 0.014 (5) |
| 340 | g_*Prevotella* | 0.075 |  | 0.146 |  |  |  |
| 341 | g_*Prevotella* |  | 0.011 (4) |  | 0.015 (4) | 0.054 (2) | 0.051 (5) |
| 342 | g_*Prevotella* |  | 0.014 (5) |  | 0.015 (4) | 0.051 (3) | 0.058 (5) |
| 343 | g_*Prevotella* |  |  | 0.102 |  |  |  |
| 344 | g_*Prevotella* |  | 0.032 (4) |  | 0.047 (4) | 0.042 (4) | 0.024 (5) |
| 345 | g_*Prevotella* |  | 0.009 (3) |  | 0.022 (4) | 0.072 (4) | 0.100 (5) |
| 346 | g_*Prevotella* | 0.008 |  | 0.240 | 0.003 (1) |  |  |
| 347 | g_*Prevotella* | 0.116 |  | 0.080 |  |  |  |
| 348 | g_*Prevotella* | 0.116 |  | 0.124 |  |  |  |
| 349 | g_*Prevotella* | 0.058 |  | 0.146 |  |  |  |
| 350 | g_*Prevotella* | 0.141 |  | 0.240 |  |  |  |
| 351 | g_*Prevotella* | 0.100 |  | 0.109 |  |  |  |
| 352 | g_*Prevotella* | 0.141 |  | 0.080 | 0.001 (1) | 0.001 (1) |  |
| 353 | g_*Prevotella* |  | 0.050 (5) |  | 0.035 (5) | 0.084 (3) | 0.044 (5) |
| 354 | g_*Prevotella* |  | 16.888 (5) |  | 13.031 (5) | 19.462 (4) | 16.010 (5) |
| 355 | g_*Prevotella* |  | 1.456 (5) |  | 4.226 (5) | 0.515 (4) | 0.807 (5) |
| 356 | g_*Prevotella* |  | 0.085 (5) |  | 0.079 (4) | 0.133 (4) | 0.041 (5) |
| 357 | g_*Prevotella* | 0.017 |  | 0.116 | 0.029 (1) |  |  |
| 358 | g_*Prevotella* | 0.174 |  | 0.320 |  |  |  |
| 359 | g_*Prevotella* | 0.124 |  | 0.007 |  |  |  |
| 360 | g_*Prevotella* |  | 0.008 (5) |  | 0.072 (4) | 0.027 (4) | 0.018 (5) |
| 361 | g_*Prevotella* | 0.008 |  | 0.226 | 0.001 (1) |  |  |
| 362 | g_*Prevotella* |  | 0.134 (5) |  | 0.126 (5) | 0.219 (4) | 0.211 (5) |
| 363 | g_*Prevotella* | 0.116 |  | 0.146 |  |  |  |
| 364 | g_*Prevotella* |  | 0.001 (3) |  | 0.007 (3) | 0.086 (4) | 0.001 (1) |
| 365 | g_*Prevotella* | 0.008 | 5.081 (5) | 0.007 | 3.117 (5) | 5.649 (4) | 6.224 (5) |
| 366 | g_*Prevotella* | 0.091 |  | 0.138 |  |  |  |
| 367 | g_*Prevotella* | 0.424 |  | 0.364 |  |  |  |
| 368 | g_*Prevotella* | 0.017 | 5.823 (5) | 0.01457 | 5.795 (5) | 13.758 (4) | 3.310 (5) |
| 369 | g_*Prevotella* | 0.058 | 0.001 (1) | 0.051 |  |  |  |
| 370 | g_*Prevotella* |  | 0.099 (5) |  | 0.060 (4) | 0.113 (4) | 0.093 (5) |
| 371 | g_*Prevotella* | 0.066 |  | 0.189 | 0.001 (1) |  |  |
| 372 | g_*Prevotella* | 0.116 |  | 0.270 |  |  |  |
| 373 | g_*Prevotella* | 0.124 |  | 0.357 | 0.001 (1) |  |  |
| 374 | g_*Prevotella* | 0.042 |  | 0.138 |  |  |  |
| 375 | g_*Prevotella* | 0.042 |  | 0.146 |  |  |  |
| 376 | g_*Prevotella* | 0.448 |  | 1.348 | 0.004 (1) |  |  |
| 377 | g_*Prevotella* | 0.033 |  | 0.109 |  |  |  |
| 378 | g_*Prevotella* | 0.548 |  | 0.138 | 0.001 (1) |  |  |
| 379 | g_*Prevotella* | 0.100 |  | 0.168 | 0.001 (1) |  |  |
| 380 | g_*Prevotella* | 1.246 |  | 1.042 | 0.001 (1) |  |  |
| 381 | g_*Prevotella* | 0.050 |  | 0.109 | 0.001 (1) |  |  |
| 382 | g_*Prevotella* | 0.124 |  | 0.248 |  |  |  |
| 383 | g_*Prevotella* | 0.100 |  | 0.233 |  |  |  |
| 384 | g_*Prevotella* | 0.033 |  | 0.146 |  |  |  |
| 385 | g_*Prevotella* | 0.141 |  | 0.240 |  |  |  |
| 386 | g_*Prevotella* | 0.033 |  | 0.095 |  |  |  |
| 387 | g_*Prevotella* | 0.108 |  | 0.080 |  |  |  |
| 388 | g_*Prevotella* | 0.141 |  | 0.080 |  |  |  |
| 389 | g_*Prevotella* | 0.100 |  | 0.146 |  |  |  |
| 390 | g_*Prevotella* | 0.307 |  | 0.087 |  |  |  |
| 391 | g_*Prevotella* | 0.042 |  | 0.102 |  |  |  |
| 392 | g_*Prevotella* | 0.008 |  | 0.124 | 0.176 (1) |  |  |
| 393 | g_*Prevotella* | 0.108 |  | 0.124 |  |  |  |
| 394 | g_*Prevotella* | 0.108 |  | 0.226 |  |  |  |
| 395 | g_*Prevotella* | 5.398 |  | 3.118 | 0.002 (1) | 0.002 (2) |  |
| 396 | g_*Prevotella* | 0.050 |  | 0.240 |  |  |  |
| 397 | g_*Prevotella* | 0.673 |  | 0.794 |  |  |  |
| 398 | g_*Prevotella* | 0.208 |  | 0.204 |  |  |  |
| 399 | g_*Prevotella* | 1.287 |  | 1.085 |  |  |  |
| 400 | g_*Prevotella* | 0.158 |  | 0.102 |  |  |  |
| 401 | g_*Prevotella* | 0.158 |  | 0.051 |  |  |  |
| 402 | g_*Prevotella* | 0.299 |  | 0.306 |  |  |  |
| 403 | g_*Prevotella* | 0.017 |  | 0.386 | 0.066 (1) | 0.002 (1) |  |
| 404 | g_*Prevotella* | 0.174 |  | 0.146 |  |  |  |
| 405 | g_*Prevotella* | 0.108 |  | 0.095 |  |  |  |
| 406 | g_*Prevotella* | 0.042 |  | 0.102 | 0.001 (1) |  |  |
| 407 | g_*Prevotella* | 1.304 |  | 1.967 |  |  |  |
| 408 | g_*Prevotella* | 0.166 | 0.001 (3) | 0.211 | 0.009 (3) | 0.001 (1) |  |
| 409 | g_*Prevotella* | 0.008 |  | 0.102 |  |  |  |
| 410 | g_*Prevotella* | 0.050 |  | 0.714 |  |  |  |
| 411 | g_*Prevotella* | 1.071 |  | 0.838 |  |  |  |
| 412 | g_*Prevotella* | 0.116 |  | 0.204 |  |  |  |
| 413 | g_*Prevotella* | 0.432 |  | 0.291 |  |  |  |
| 414 | g_*Prevotella* | 0.133 |  | 0.116 |  |  |  |
| 415 | g_*Prevotella* | 0.241 |  | 0.342 |  |  |  |
| 416 | g_*Prevotella* | 0.116 |  | 0.102 | 0.001 (1) | 0.011 (1) |  |
| 417 | g_*Prevotella* | 0.042 | 0.003 (5) | 0.008 | 0.002 (2) | 0.008 (2) |  |
| 418 | g_*Prevotella* | 0.432 |  | 0.670 | 0.001(1) |  |  |
| 419 | g_*Prevotella* | 0.390 |  | 0.350 |  |  |  |
| 420 | g_*Prevotella* | 0.124 | 0.001 (1) | 0.102 |  |  |  |
| 421 | g_*Prevotella* | 0.066 |  | 0.066 |  |  |  |
| 422 | g_*Prevotella* | 0.124 |  | 0.160 | 0.001 (1) |  |  |
| 423 | g_*Prevotella* | 0.091 |  | 0.102 |  |  |  |
| 424 | g_*Prevotella* | 0.033 |  | 0.284 | 0.070 (1) |  |  |
| 425 | g_*Prevotella* |  | 0.323 (5) |  | 0.557 (5) | 0.875 (4) | 0.547 (5) |
| 426 | g_*Prevotella* | 0.042 |  |  |  |  |  |
| 427 | g_*Prevotella* | 0.042 |  | 0.175 |  |  |  |
| 428 | g_*Prevotella* | 0.042 |  | 0.124 |  |  |  |
| 429 | g_*Prevotella* | 0.498 |  | 0.291 |  |  |  |
| 430 | g_*Prevotella* |  | 1.831 (5) |  | 0.343 (5) | 1.111 (4) | 1.422 (5) |
| 431 | g_*Prevotella* | 0.075 |  | 0.182 |  |  |  |
| 432 | g_*Prevotella* | 0.274 |  | 0.014 |  |  |  |
| 433 | g_*Prevotella* | 0.050 |  | 0.036 |  |  |  |
| 434 | g_*Prevotella* | 0.108 |  | 0.029 |  |  |  |
| 435 | g_*Prevotella* |  | 0.322 (5) |  | 3.771 (5) | 1.142 (4) | 1.230 (5) |
| 436 | g_*Prevotella* | 0.232 |  | 0.211 | 0.004 (1) |  |  |
| 437 | g_*Prevotella* | 0.050 |  | 0.080 |  |  |  |
| 438 | g_*Prevotella* |  | 2.615 (5) |  | 2.353 (5) | 12.800 (4) | 11.654 (5) |
| 439 | g_*Prevotella* | 0.208 |  | 0.073 |  |  |  |
| 440 | g_*Prevotella* | 0.714 |  | 0.175 |  |  |  |
| 441 | g_*Prevotella* | 0.042 |  | 0.299 | 0.001 (1) |  |  |
| 442 | g_*Prevotella* | 0.208 | 0.001 (1) | 0.226 |  |  | 0.001 (1) |
| 443 | g_*Prevotella* | 0.266 |  | 0.510 |  |  |  |
| 444 | g_*Prevotella* | 0.124 |  | 0.036 |  |  |  |
| 445 | g_*Prevotella* | 0.174 |  | 0.051 |  |  |  |
| 446 | g_*Prevotella* |  |  |  |  |  |  |
| 447 | g_*Prevotella* | 3.048 |  | 4.320 |  | 0.002 (1) |  |
| 448 | g_*Prevotella* | 0.747 |  | 0.488 |  |  |  |
| 449 | g_*Prevotella* | 0.232 |  | 0.175 |  |  |  |
| 450 | g_*Prevotella* | 0.141 |  | 0.255 |  |  |  |
| 451 | g_*Prevotella* | 0.199 |  | 0.044 |  |  |  |
| 452 | g_*Prevotella* | 0.124 |  | 0.197 |  |  |  |
| 453 | g_*Prevotella* | 0.116 |  | 0.109 |  |  |  |
| 454 | g_*Prevotella* | 0.390 |  | 0.168 |  |  |  |
| 455 | g_*Prevotella* | 0.108 |  | 0.029 |  |  |  |
| 456 | g_*Prevotella* | 0.158 |  | 0.3861 |  |  |  |
| 457 | g_*Prevotella* | 0.224 | 0.001 (1) | 0.262 | 0.119 (1) | 0.001 (1) |  |
| 458 | g_*Prevotella* | 0.083 |  | 0.109 |  |  |  |
| 459 | g_*Prevotella* | 0.158 |  | 0.306 |  |  |  |
| 460 | g_*Prevotella* | 0.141 |  | 0.146 |  |  |  |
| 461 | g_*Prevotella* | 0.490 |  | 0.612 |  |  |  |
| 462 | g_*Prevotella* | 0.365 | 0.001 (2) | 0.284 | 0.062 (4) | 0.078 (1) |  |
| 463 | g_*Prevotella* | 0.100 |  | 0.095 |  |  |  |
| 464 | g_*Prevotella* | 0.042 |  | 0.051 | 0.003 (1) |  |  |
| 465 | g_*Prevotella* | 0.415 |  | 0.138 |  |  |  |
| 466 | g_*Prevotella* | 0.108 |  | 0.087 |  |  |  |
| 467 | g_*Prevotella* | 1.055 | 0.001 (1) | 0.510 | 0.001 (1) | 0.028 (2) |  |
| 468 | g_*Prevotella* | 0.399 |  | 0.393 |  |  |  |
| 469 | g_*Prevotella* | 0.100 |  | 0.159 |  |  |  |
| 470 | g_*Prevotella* | 1.345 |  | 1.472 | 0.001 (1) |  |  |
| 471 | g_*Prevotella* | 0.050 |  | 0.153 |  |  |  |
| 472 | g_*Prevotella* |  | 1.758 (5) |  | 2.263 (5) | 2.825 (4) | 2.325 (5) |
| 473 | g_*Prevotella* | 0.050 |  | 0.153 |  |  |  |
| 474 | g_*Prevotella* | 0.349 |  | 0.248 | 0.001 (1) |  |  |
| 475 | g_*Prevotella* |  | 0.063 (4) | 0.015 | 0.308 (3) | 0.097 (4) | 0.064 (4) |
| 476 | g_*Prevotella* |  | 0.008 (5) |  | 0.073 (4) | 0.014 (4) | 0.001 (2) |
| 477 | g_*Pseudoramibacter*_*Eubacterium* | 0.490 |  | 0.029 |  |  |  |
| 478 | g_*Ruminobacter* | 0.232 |  | 0.014 |  | 0.001 (1) |  |
| 479 | g_*Ruminobacter* |  | 0.026 (1) |  |  | 0.008 (2) | 0.002 (1) |
| 480 | g_*Ruminococcus* |  | 0.072 (1) |  |  | 0.023 (2) | 0.006 (1) |
| 481 | g_*Ruminococcus* |  | 0.068 (1) |  |  | 0.040 (2) | 0.010 (1) |
| 482 | g_*Ruminococcus* |  | 0.034 (1) |  |  | 0.007 (2) | 0.002 (1) |
| 483 | g_*Ruminococcus* | 0.174 |  | 0.095 |  |  |  |
| 484 | g_*Ruminococcus* | 0.058 |  | 0.379 |  |  |  |
| 485 | g_*Ruminococcus* | 0.183 |  | 0.153 |  |  |  |
| 486 | g_*Ruminococcus* |  | 3.344 (1) |  | 0.001 (1) | 0.906 (2) | 0.202 (1) |
| 487 | g_*Ruminococcus* |  | 0.025 (1) | 0.007 |  | 0.018 (2) | 0.007 (1) |
| 488 | g_*Ruminococcus* | 0.042 | 0.006 (1) | 0.014 | 0.093 (5) | 0.135 (2) |  |
| 489 | g_*Ruminococcus* |  | 2.208 (4) |  | 0.277 (4) | 0.872 (3) | 0.503 (4) |
| 490 | g_*Shuttleworthia* |  | 0.008 (5) |  | 0.053 (3) | 0.020 (4) | 0.013 (5) |
| 491 | g_*Shuttleworthia* |  | 0.068 (5) |  | 0.005 (2) | 0.030 (4) | 0.042 (4) |
| 492 | g_*Shuttleworthia* |  | 0.011 (4) |  | 0.007 (5) | 0.043 (4) | 0.008 (4) |
| 493 | g_*Shuttleworthia* |  | 0.015 (4) |  | 0.056 (4) | 0.011 (4) | 0.001 (3) |
| 494 | g_*Shuttleworthia* |  | 0.042 (3) |  | 0.009 (2) | 0.051 (3) | 0.007 (4) |
| 495 | g_*Shuttleworthia* |  | 0.092 (4) |  | 0.056 (5) | 0.188 (4) | 0.090 (5) |
| 496 | g_*Shuttleworthia* |  | 0.034 (5) |  | 0.029 (3) | 0.026 (4) | 0.029 (5) |
| 497 | g_*Shuttleworthia* |  | 0.005 (4) |  | 0.028 (3) | 0.017 (4) | 0.005 (5) |
| 498 | g_*Shuttleworthia* |  | 0.028 (4) |  | 0.006 (2) | 0.028 (3) | 0.005 (4) |
| 499 | g_*Shuttleworthia* |  | 0.004 (4) |  | 0.015 (3) | 0.057 (4) | 0.013 (5) |
| 500 | g_*Shuttleworthia* |  | 0.001 (3) |  | 0.002 (2) | 0.111 (3) | 0.002 (2) |
| 501 | g_*Shuttleworthia* |  | 0.007 (4) |  | 0.016 (4) | 0.062 (4) | 0.014 (3) |
| 502 | g_*Shuttleworthia* |  | 0.002 (3) |  | 0.059 (3) | 0.066 (2) | 0.007 (1) |
| 503 | g_*Shuttleworthia* |  | 0.016 (5) |  | 0.032 (5) | 0.098 (4) | 0.014 (5) |
| 504 | g_*Shuttleworthia* |  | 0.003 (5) |  | 0.062 (3) | 0.044 (3) | 0.008 (4) |
| 505 | g_*Shuttleworthia* |  | 0.014 (5) |  | 0.049 (4) | 0.030 (4) | 0.014 (5) |
| 506 | g_*Shuttleworthia* |  | 0.026 (5) |  | 0.050 (5) | 0.095 (4) | 0.035 (5) |
| 507 | g_*Shuttleworthia* |  | 0.011 (4) |  | 0.031 (3) | 0.063 (3) | 0.013 (3) |
| 508 | g_Shuttleworthia | 0.174 |  | 0.080 |  |  |  |
| 509 | g_*Shuttleworthia* |  | 0.009 (4) |  | 0.001 (1) | 0.090 (1) | 0.001 (2) |
| 510 | g_*Shuttleworthia* | 0.008 | 2.834 (5) | 0.007 | 5.290 (5) | 4.926 (4) | 2.017 (5) |
| 511 | g_*Shuttleworthia* |  | 0.031 (2) |  |  | 0.010 (2) | 0.002 (1) |
| 512 | g_*Succiniclasticum* | 0.282 | 0.001 (2) | 0.677 | 0.001 (2) | 0.002 (3) | 0.001 (2) |
| 513 | g_*Succiniclasticum* | 0.108 |  | 0.066 |  |  |  |
| 514 | g_*Succiniclasticum* | 1.162 | 0.002 (2) | 2.171 | 0.001 (3) | 0.004 (2) | 0.001 (3) |
| 515 | g_*Succiniclasticum* |  | 0.027 (4) |  | 0.006 (2) | 0.008 (3) | 0.010 (3) |
| 516 | g_*Succinivibrio* | 0.236 |  | 0.066 |  |  |  |
| 517 | g_*Succinivibrio* | 0.091 | 0.001 (2) | 0.007 |  |  |  |
| 518 | g_*Succinivibrio* | 0.025 |  | 0.007 |  |  |  |
| 519 | g_*Succinivibrio* | 0.108 |  | 0.007 |  |  |  |
| 520 | g_*Succinivibrio* | 0.050 | 0.001 (1) | 0.051 | 0.001 (1) | 0.026 (1) |  |
| 521 | g_*Succinivibrio* | 0.008 |  | 0.007 |  | 0.027 (1) |  |
| 522 | g_*Treponema* | 0.124 | 0.001 (1) | 0.066 |  | 0.059 (2) | 0.001 (1) |
| 523 | g_*Treponema* | 0.249 | 0.001 (1) | 0.138 |  | 0.016 (2) |  |
| 524 | g_*Treponema* | 0.374 |  | 0.087 |  |  |  |
| 525 | g_*Treponema* | 0.066 |  | 0.007 |  | 0.160 (2) |  |
| 526 | g_*Treponema* |  |  | 0.014 |  | 0.100 (2) |  |
| 527 | g_*Treponema* | 0.033 |  | 0.014 | 0.001 (1) | 0.175 (2) |  |
| 528 | g_*Treponema* | 0.108 |  |  |  |  |  |
| 529 | g_*Treponema* | 0.174 |  | 0.073 |  |  |  |
| 530 | g_*Treponema* | 0.124 |  | 0.080 |  |  |  |
| 531 | g_*Treponema* | 0.050 |  | 0.116 |  |  |  |
| 532 | g_YRC22 | 0.033 |  | 0.007 |  |  |  |
| 533 | g_YRC22 | 0.008 |  | 0.116 |  |  |  |
| 534 | g_YRC22 | 0.390 |  | 0.102 |  |  |  |
| 535 | s_*Fibrobacter* *succinogenes* | 0.756 | 0.001 (2) | 0.328 | 0.001 (1) | 0.006 (2) | 0.001 (1) |
| 536 | s_*Fibrobacter* *succinogenes* | 0.149 |  | 0.080 |  |  |  |
| 537 | s_*Fibrobacter* *succinogenes* | 0.257 |  | 0.036 |  |  |  |
| 538 | s_Fibrobacter *succinogenes* | 0.025 |  | 0.022 |  |  |  |
| 539 | s_*Prevotella* *copri* |  | 0.004 (4) |  | 0.004 (3) | 0.031 (2) | 0.049 (5) |
| 540 | s_*Prevotella* *copri* |  | 0.027 (5) |  | 0.017 (4) | 0.137 (3) | 0.022 (5) |
| 541 | s_*Prevotella* *copri* |  | 0.011 (5) |  | 0.022 (4) | 0.303 (4) | 0.008 (5) |
| 542 | s_*Prevotella* *copri* | 0.158 |  | 0.102 |  | 0.001 (1) |  |
| 543 | s_*Prevotella* *melaninogenica* |  | 0.114 (5) |  | 0.036 (5) | 0.050 (4) | 0.086 (4) |
| 544 | s_*Pyramidobacter* *piscolens* | 0.731 |  | 0.889 |  | 0.001 (1) |  |
| 545 | s_*Selenomonas* *ruminantium* |  | 0.008 (4) |  | 0.024 (3) | 0.059 (3) | 0.010 (3) |
| 546 | s_*Sharpea* p-3329-23G2 |  | 0.005 (4) |  | 0.030 (3) | 0.017 (4) | 0.008 (4) |

* OTUs were clustered using open OTU picking using QIIME2 .

^⁑^ Inoc-A and Inoc-B were inoculated with fresh rumen fluid from donor A and donor B cows, respectively, while Ctrl-A and Ctrl-B were drenched with autoclaved rumen fluid from donor C and donor D cows, respectively. Because the rumen fluid from these two cows were autoclaved, the rumen microbiota sequence data were not included. All the recipient groups had 5 calves except C, which lost one calf. Values in parentheses indicate the number of calves that had the OTUs detected.

^⁂^ The lowest taxon to which the OTUs can be assigned to.

OTUs are shown in blue when found in both donor-A and Inoc-A, in red when found in both donor-B and Inoc-B, and in green when found in donor-A, donor-B, Inoc-A, and Inoc-B.
