# Supplementary figures and images for: Repeated Inoculation of Young Calves With Rumen Microbiota Does Not Significantly Modulate the Rumen Prokaryotic Microbiota Consistently but Decreases Diarrhea
Source: Front Microbiol. 2020 Jun 24;11:1403. doi: 10.3389/fmicb.2020.01403 (PMC7326819; doi:10.3389/fmicb.2020.01403)

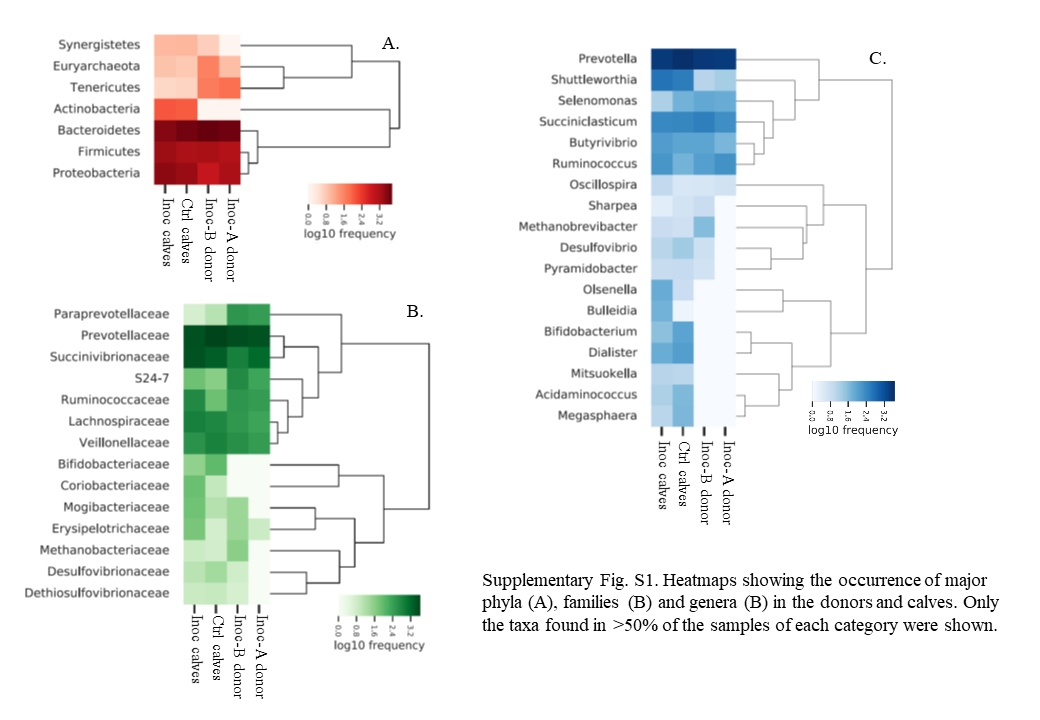

Supplement: Supplementary file 2 [file Image_1.TIF]

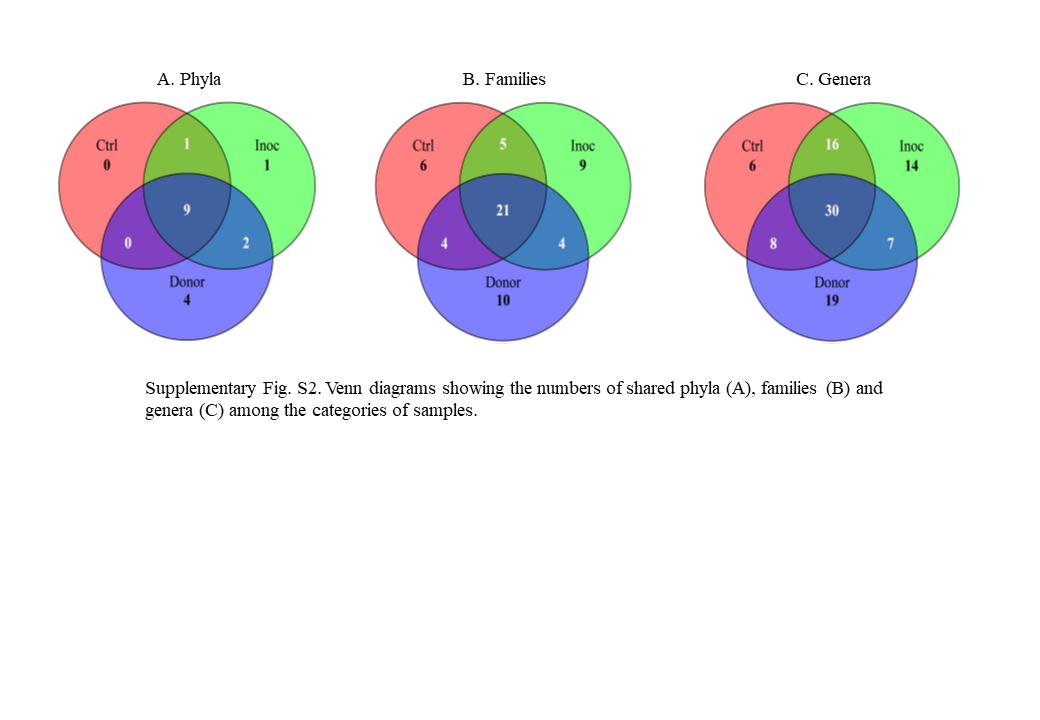

Supplement: Supplementary file 3 [file Image_2.TIF]

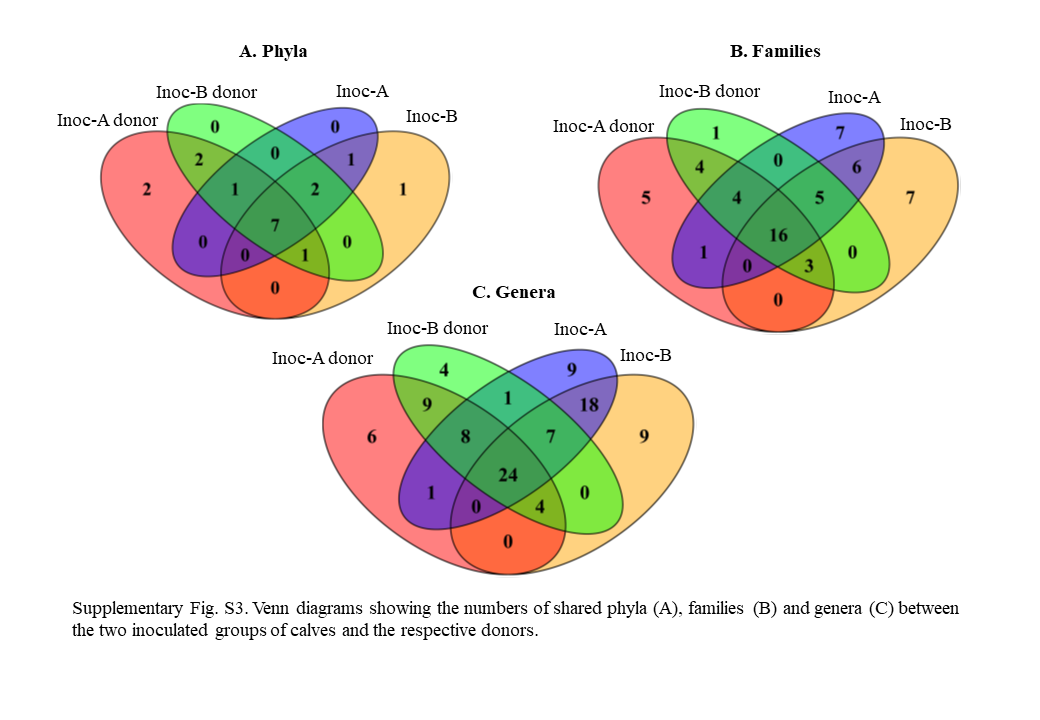

Supplement: Supplementary file 4 [file Image_3.TIF]

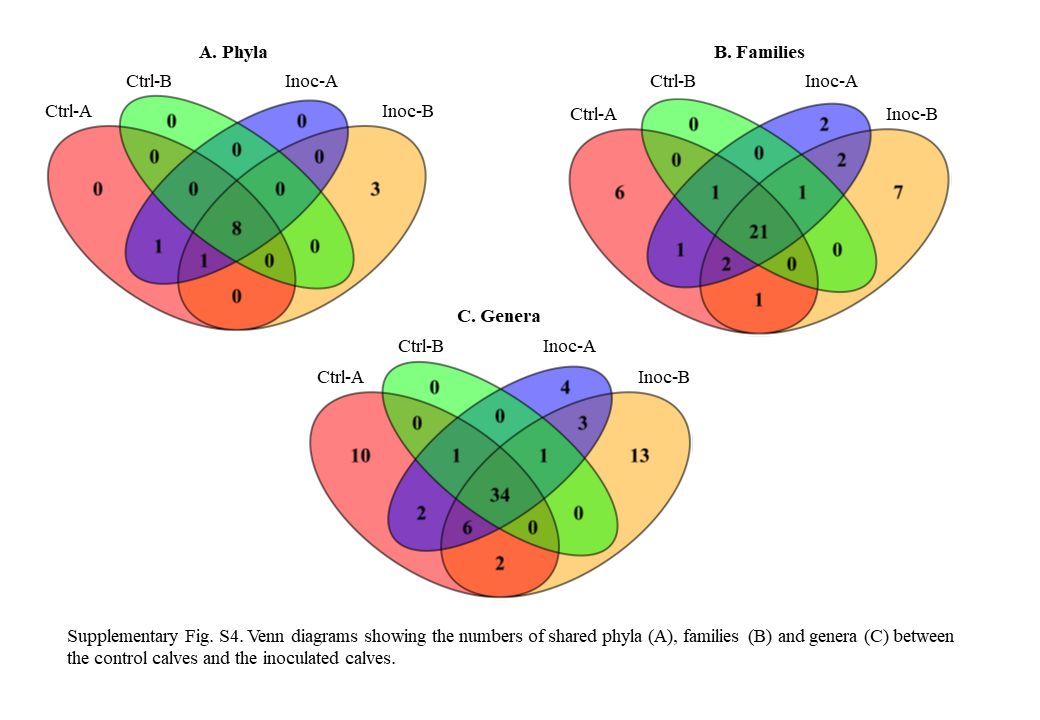

Supplement: Supplementary file 5 [file Image_4.TIF]

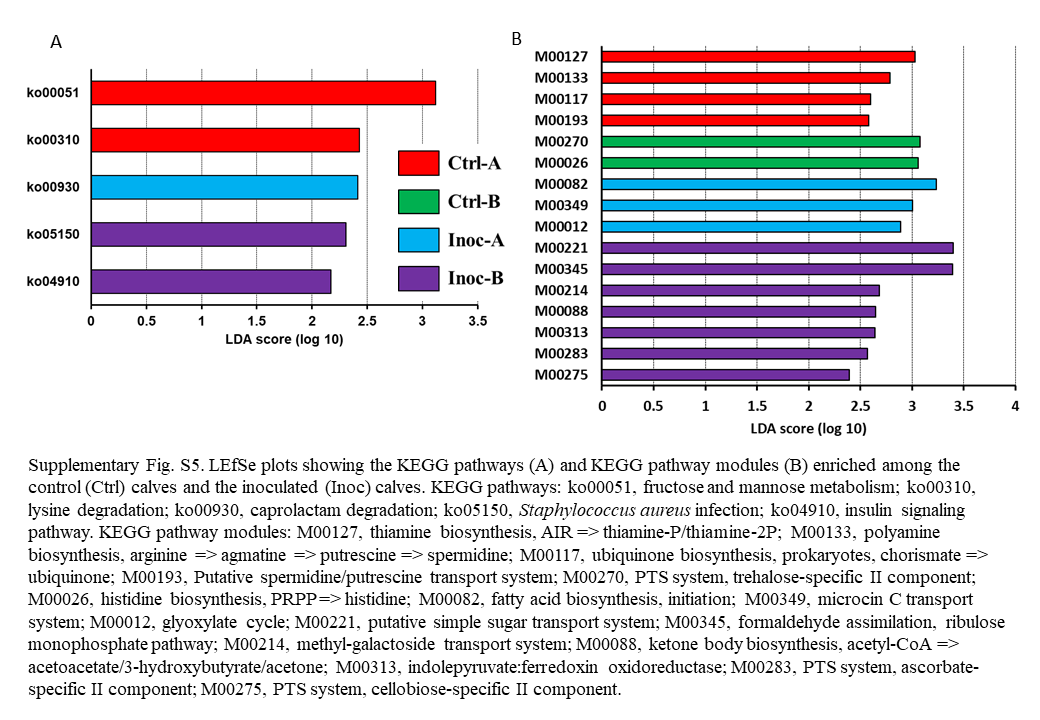

Supplement: Supplementary file 6 [file Image_5.TIF]
